# Polymorphism and pseudosymmetry of 10,10′-oxybis(9-thia-10-hydro-10-boraanthracene)

**DOI:** 10.1107/S205698901900522X

**Published:** 2019-04-25

**Authors:** Julian Radtke, Hans-Wolfram Lerner, Michael Bolte

**Affiliations:** a Institut für Anorganische und Analytische Chemie, Goethe-Universität Frankfurt, Max-von-Laue-Strasse 7, 60438 Frankfurt am Main, Germany

**Keywords:** crystal structure, twinning, pseudo-symmetry, polymorphism

## Abstract

We have encountered two polymorphs of the title compound, C_24_H_16_B_2_OS_2_, both of which display almost the same unit-cell parameters. Compound (I) crystallizes in the non-centrosymmetric space group *P*2_1_ with four mol­ecules in the asymmetric unit. These mol­ecules are related by pseudosymmetry. As a result, the space group looks like *P*2_1_/*c*, but the structure cannot be refined successfully in that space group. Compound (II) on the other hand crystallizes in the centrosymmetric space group *P*2_1_/*c* with only two mol­ecules in the asymmetric unit. The crystals studied for (I) and (II) were both non-merohedral twins.

## Abstract 2

Two polymorphs of the title compound are reported. The first crystallizes in the non-centrosymmetric space group *P*2_1_ with four mol­ecules, related by pseudosymmetry, in the asymmetric unit. The second crystallizes in the centrosymmetric space group *P*2_1_/*c* with only two mol­ecules in the asymmetric unit.

## Chemical context   

Extended, conjugated π-systems are of great inter­est for both fundamental as well as applied materials research. They are typically utilized as key components for optoelectronic devices, such as organic field-effect transistors (OFETs), organic light-emitting diodes (OLEDs), and organic photovoltaics (OPVs), or as electrode materials in lithium batteries, often acting as the functional layer within these devices (Wu *et al.*, 2007 ▸; Harvey, 1997 ▸). Archetypal examples from this class of compounds are polycyclic aromatic hydro­carbons (PAHs), which can be considered as well-defined cutouts of graphene with adjustable mol­ecular structure design and predictable supra­molecular arrangements (Rieger & Müllen, 2010 ▸). The properties of these all-carbon compounds can be further improved by substitutional doping with main-group elements, which has long been known for elements such as nitro­gen, phospho­rous, or sulfur (Stępień *et al.*, 2017 ▸). However, only a few boron-doped PAHs have been synthesized to date, mainly because of synthetic difficulties (von Grotthuss *et al.*, 2018 ▸).
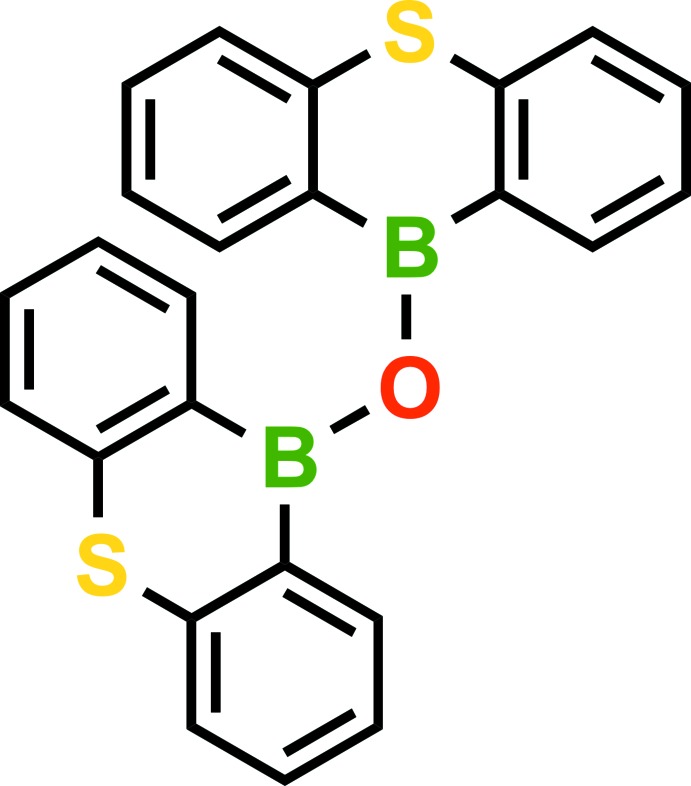


The three-coordinate boron centre can be regarded as an equivalent to a carbenium ion due to its vacant *p*_z_ orbital, and therefore acts as both a π-electron acceptor and σ-electron donor. The combination of electron-deficient boron centres with pnictogens or chalcogens, respectively, leads to compounds isoelectronic to the all-carbon systems (Liu & Marder, 2008 ▸). Thus, we set out to design extended, conjugated π-systems, which also incorporate sulfur atoms in addition to boron centres.

## Structural commentary   

**Figure 1:**
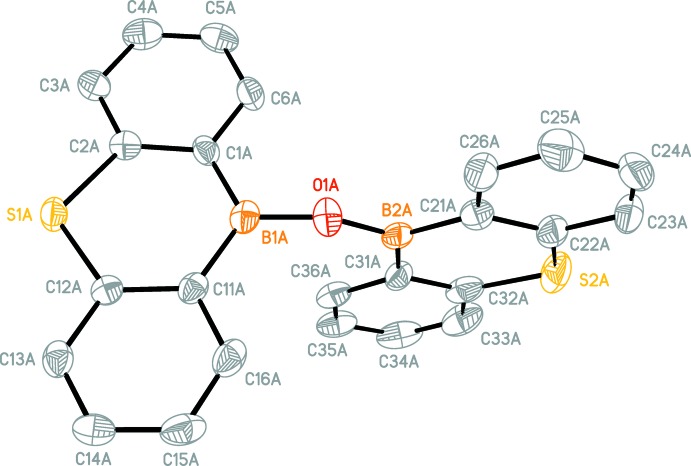
A perspective view of one mol­ecule in the asymmetric unit of (I). H atoms are omitted for clarity.

Mol­ecules of the title compounds (Figs. 1 ▸–4 ▸ ▸ ▸) feature two 9-thia-10-bora-anthracene moieties connected by an oxygen bridge. The dihedral angles between the two 9-thia-10-bora-anthracene moieties in (I) are 86.26 (6)° for mol­ecule *A*, 88.07 (5)° for mol­ecule *B*, 85.82 (5)° for mol­ecule *C*, 89.69 (6)° for mol­ecule *D*.

The dihedral angles between the two 9-thia-10-bora-anthracene moieties in (II) are 84.03 (9)° for mol­ecule 1 and 87.19 (9)° for mol­ecule *A*.

Mol­ecules *A* and *C* of (I) show a similar dihedral angle as mol­ecule 1 of (II) and mol­ecules *B* and *D* of (I) have a dihedral angle close to the one of mol­ecule *A* of (II).

The B—O—B angles are significantly widened in both structures [(I): B2*A*—O1*A*—B1*A* 146.0 (6)°, B1*B*—O1*B*—B2*B* 164.8 (7)°, B2*C*—O1*C*—B1*C* 166.0 (7)°, B1*D*—O1*D*—B2*D* 165.9 (7)°; (II): B2—O1—B1 172.0 (7)° B1*A*—O1*A*—B2*A* 159.5 (7)°].

## Supra­molecular features   

**Figure 5:**
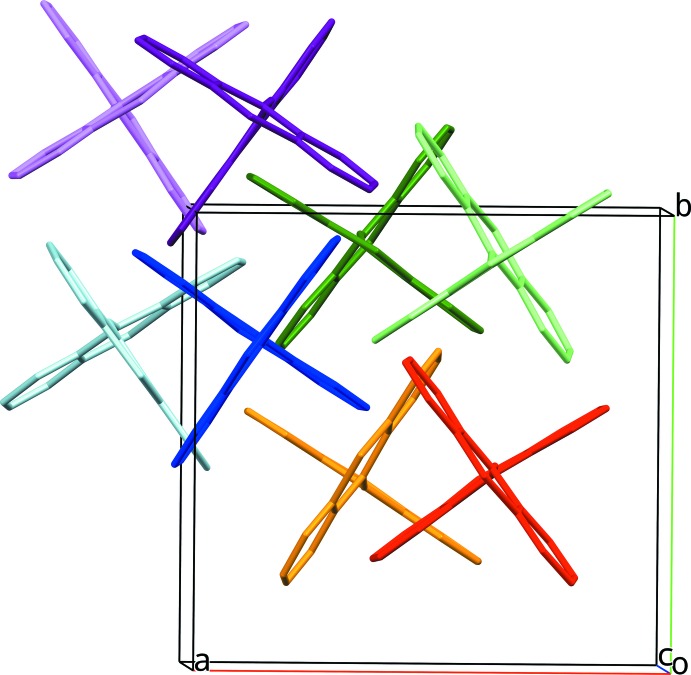
Crystal packing of (I). Mol­ecules occupying similar positions in (I) and (II) are drawn with the same colour to illustrate the similarities between (I) and (II).

The mol­ecular packing in both structures is very similar. In Figs. 5 ▸ and 6 ▸, mol­ecules that occupy a similar position in both structures are drawn with the same colour in order to illustrate the similarities between the structures.

**Figure 7:**
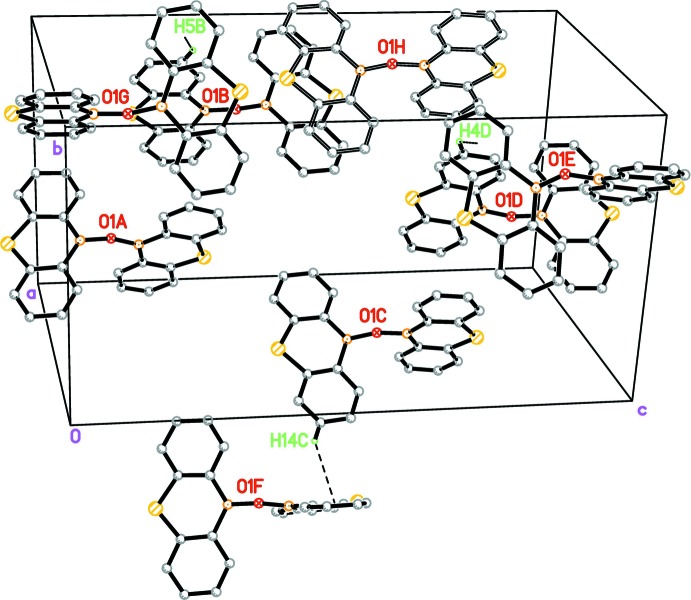
Partial packing diagram of (I). The mol­ecules containing O1*A*, O1*B*, O1*C* and O1*D* are the four symmetry-independent mol­ecules in the asymmetric unit. The mol­ecule containing O1*E* was generated by the symmetry operator 1 − *x*, 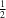 + *y*, 1 − *z*, that containing O1*F* by the symmetry operator 1 − *x*, −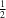 + *y*, 1 − *z*, that containing O1*G* by the symmetry operator 1 − *x*, 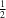 + *y*, 1 − *z* and that containing O1*H* by the symmetry operator *x*, 1 + *y*, *z*. This mol­ecule is drawn with open bonds in order to show the π–π inter­action. Only the H atoms involved in a C—H⋯π inter­action are shown and labelled.

In (I), there are three C—H⋯π inter­actions with H⋯centroid (*Cg*) distances less than 3.0 Å [C4*D*—H4*D*⋯*Cg*(C21*A*–C26*A*)^i^: H⋯*Cg* = 2.90 Å, C—H⋯*Cg =* 137°; C14*C*—H14*C*⋯*Cg*(C1*C*–C6*C*)^ii^: H⋯*Cg* = 2.93 Å, C—H⋯*Cg* = 139°; C5*B*—H5*B*⋯*Cg*(C1*D*–C6*D*)^iii^: H⋯*Cg* = 2.78 Å, C—H⋯*Cg* = 139°; symmetry codes: (i) 1 − *x*, 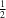 + *y*, 1 − *z*; (ii) 1 − *x*, −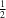 + *y*, 1 − *z*; (iii) 1 − *x*, 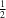 + *y*, 1 − *z*] and there is a π–π inter­action between the thia-boraanthracene fragment of mol­ecule *B* with the thia-boraanthracene fragment of mol­ecule *C* (transformed by *x*, *y* + 1, *z*) with an approximate distance of 3.8 Å between the thia-boraanthracene planes (Fig. 7 ▸).

**Figure 8:**
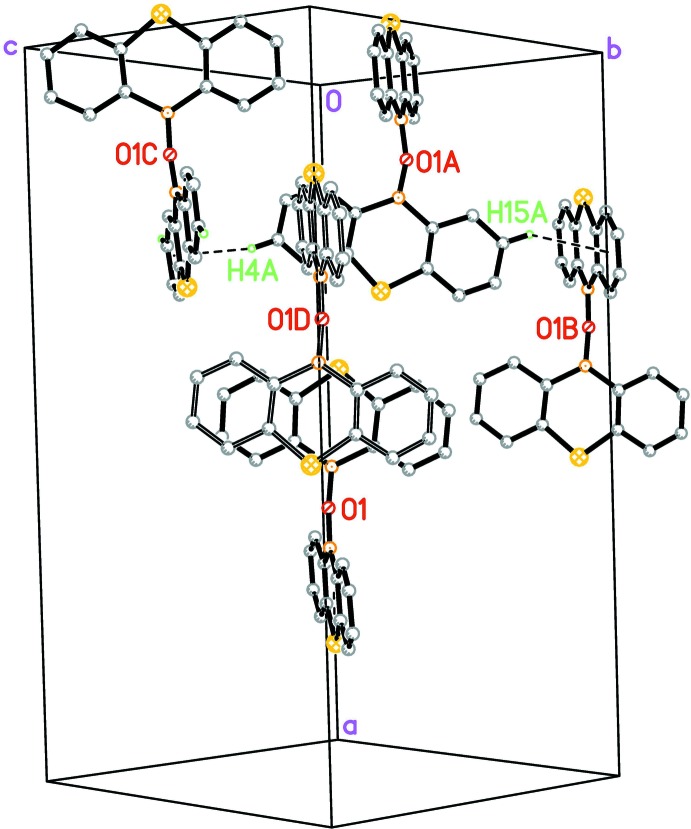
Partial packing diagram of (II). The mol­ecules containing O1 and O1*A* are the two symmetry-independent mol­ecules in the asymmetric unit. The mol­ecule containing O1*B* was generated by the symmetry operator *x*, 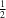 − *y*, 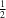 + *z*, that containing O1*C* by the symmetry operator 1 − *x*, 1 − *y*, −*z* and that containing O1*D* by the symmetry operator 1 − *x*, 1 − *y*, 1 − *z*. This mol­ecule is drawn with open bonds in order to show the π–π inter­action. Only the H atoms involved in a C—H⋯π inter­action are shown and labelled.

In (II), there are two C—H⋯π inter­actions with H⋯*Cg* less than 3.0 Å [C4*A*—H4*A*⋯*Cg*(C11*A*–C16*A*)^i^: H⋯*Cg =* 2.77 Å, C—H⋯*Cg =* 147°; C15*A*—H15*A*⋯*Cg*(C31–C36)^ii^: H⋯*Cg =* 2.91 Å, C—H⋯*Cg =* 138°; symmetry codes: (i) *x*, 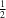 − *y*, 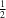 + *z*; (ii) 1 − *x*, 1 − *y*, −*z*] and there is a π–π inter­action between two thia-boraanthracene fragment related by a centre of inversion (−*x* + 1, −*y* + 1, −*z* + 1) with an approximate distance of 3.8 Å between the thia-boraanthracene planes (Fig. 8 ▸).

## Database survey   

There are structures in the CSD (version 5.40 of November 2018; Groom *et al.*, 2016 ▸) in which the thia-boraanthracene scaffold is comparable with the title compound, namely 2,9-di-*t*-butyl-7,14-dimesityl-7,14-di­hydro-5,12-di­thia-7,16-dibora­penta­cene (LIPFAS; Agou *et al.*, 2007 ▸), 10-mesityl-10*H*-9-thia-10-boraanthracene (LIPFEW; Agou *et al.*, 2007 ▸) and 10-mesityl-2,3,7,8-tetra­meth­oxy-10*H*-pheno­thia­borine (QONKAG; Kobayashi *et al.*, 2009 ▸). The B—C and S—C bond lengths as well as the C—B—C and C—S—C bond angles do not vary markedly between (I), (II) and the three comparable structures retrieved from the CSD (see Table 1 ▸).

## Synthesis and crystallization   

10-Bromo-9-thia-10-hydro-10-borananthracene was synthesized according to a literature known procedure (Solé & Gabbaï, 2004 ▸). 2-Amino­ethanol (60 mg, 0.98 mmol) was added to a solution of 10-bromo-9-thia-boraanthracene (90 mg, 0.33 mmol) in 10 mL of toluene at room temperature. The yellow solution turned colorless overnight and a fluffy precipitate formed. The solvent was removed and the obtained white solid was dissolved in 5 mL of ethanol without further purification. 1 mL of 6*N* HCl was added to this clear solution at 273 K, after which the reaction mixture turned opaque, further addition of 5 mL of 1*N* HCl caused the formation of a voluminous precipitate. After stirring for 90 minutes at 273 K, the reaction mixture was filtered, and the residue was washed with ice-cold water (3 × 3 mL). The remaining white solid was dried under vacuum at 333 K to remove traces of water and was afterwards sublimated under vacuum at 503 K to give 10,10′-oxybis[9-thia-10-hydro-10-boraanthracene] as crystalline material. Yield: 53 mg (0.13 mmol, 80%).

Note: Crystals of polymorph (I) were obtained *via* slow evaporation of a saturated solution of 10,10′-oxybis[9-thia-10-hydro-10-boraanthracene] in CH_2_Cl_2_ at room temperature. Crystals of polymorph (II) were obtained *via* sublimation of 10,10′-oxybis[9-thia-10-hydro-10-boraanthracene].

**^1^H NMR (500.18 MHz, CD_2_Cl_2_):** δ = 8.02 (*dd*, ^3^ *J*_H-H_ = 7.6 Hz, ^4^ *J*_H-H_ = 0.8 Hz, 2H, H-4,5), 7.69 (*d*, ^3^ *J*_H-H_ = 8.0 Hz, 2H, H-1,8), 7.60–7.55 (*m*, 2H, H-2,7), 7.25 ppm (*t*, ^3^ *J*_H-H_ = 7.5 Hz, 2H, H-3,6).

**^13^C{^1^H} NMR (125.78 MHz, CD_2_Cl_2_):** δ = 125.2 (C-3,6), 126.0 (C-1,8), 129.0 (C-4a,10a), 132.1 (C-2,7), 135.0 (C-4,5), 145.9 ppm (C-8a,9a)

**^11^B NMR (160.5 MHz, CD_2_Cl_2_):** δ = 39.2 ppm

**EA (%):** Calculated for C_24_H_16_B_2_OS_2_ [406.14]: C 70.98, H 3.97, S 15.79; found: C 70.14, H 4.09, S 16.06

## Refinement   

Crystal data, data collection and structure refinement details are summarized in Table 2 ▸. The H atoms for both structures were refined using a riding model with C—H = 0.95 Å and with *U*_iso_(H) = 1.2*U*_eq_(C). The fractional contribution of the minor twin domain refined to 0.4753 (7) for (I) and 0.4547 (15) for (II). The absolute structure of (I) could not be reliably determined, the Flack x parameter (Parsons *et al.*, 2013 ▸) refined to 0.28 (3).

It is remarkable that (I) and (II) show almost the same cell parameters. The *a* and *c* axes of (II) were chosen in order to refine the structure in the conventional setting for *P*2_1_/*c*. Compound (I) crystallizes in the monoclinic non-centrosymmetric space group *P*2_1_ with four mol­ecules in the asymmetric unit. A search for higher symmetry with the program *PLATON* (Spek, 2009 ▸) reveals that 97% of the structure complies with a centre of inversion and *PLATON* suggests a space group change to *P*2_1_/*c*. However, the structure cannot be refined successfully in this space group. The displacement parameters of two atoms go NPD and the figures of merit are rather bad (*R*_1_ = 0.339, *wR*_2_ = 0.722). On the other hand, compound (II) can be refined in the centrosymmetric space group *P*2_1_/*c* with just two mol­ecules in the asymmetric unit. A closer look at the systematic absence exceptions for (I) (Table 3 ▸) and (II) (Table 4 ▸) reveals a remarkable difference between the two. For (I), the reflections which should be extinct for an *a* glide plane are weaker than those for a *c* or an *n* glide plane. Nevertheless, they are definitely observed. For (II), on the other hand, the reflections which should be extinct for a *c* glide plane are doubtlessly extinct. In (II), the dis­place­ment ellipsoids of some C atoms in the outer positions of the aromatic rings are enlarged, which is most probably due to slight disorder. This disorder can be explained by a rotation of the thia-boraanthracene moiety about an axis perpendicular to the mean plane of this fragment running through the center of the central ring.

## Supplementary Material

Crystal structure: contains datablock(s) I, II, global. DOI: 10.1107/S205698901900522X/lh5900sup1.cif

Structure factors: contains datablock(s) I. DOI: 10.1107/S205698901900522X/lh5900Isup2.hkl

Structure factors: contains datablock(s) II. DOI: 10.1107/S205698901900522X/lh5900IIsup3.hkl

Supporting information file. DOI: 10.1107/S205698901900522X/lh5900Isup4.cml

NMR spectrum 1. DOI: 10.1107/S205698901900522X/lh5900sup5.pdf

NMR spectrum 2. DOI: 10.1107/S205698901900522X/lh5900sup6.pdf

NMR spectrum 3. DOI: 10.1107/S205698901900522X/lh5900sup7.pdf

CCDC references: 1910372, 1910371

Additional supporting information:  crystallographic information; 3D view; checkCIF report

## Figures and Tables

**Figure 2:**
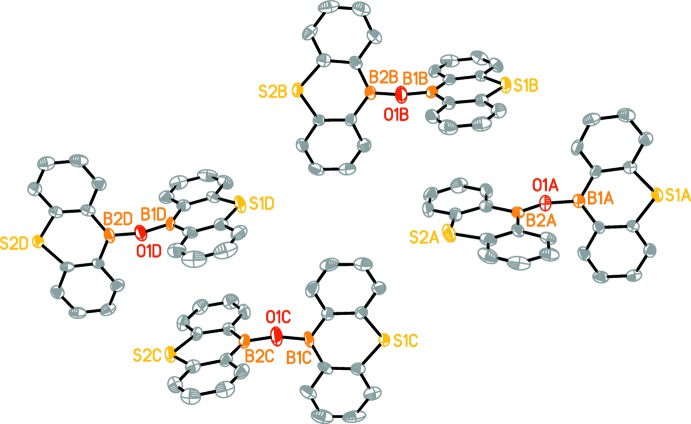
A perspective view of the four mol­ecules in the asymmetric unit of (I). H atoms are omitted for clarity. Only B, O and S atoms are labelled.

**Figure 3:**
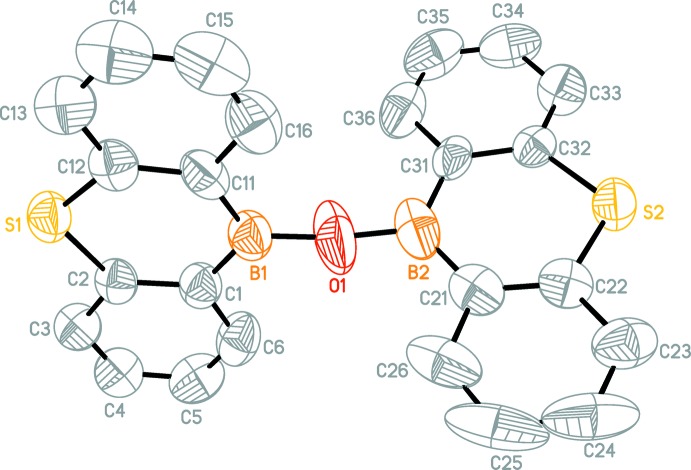
A perspective view of one mol­ecule in the asymmetric unit of (II). H atoms are omitted for clarity.

**Figure 4:**
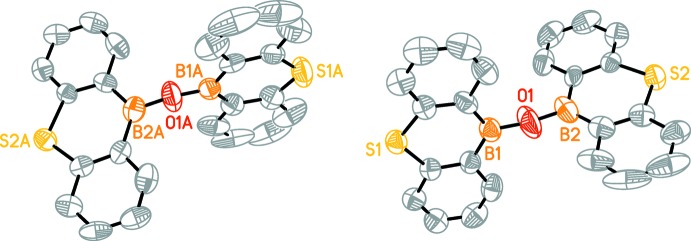
A perspective view of both mol­ecules in the asymmetric unit of (II). H atoms are omitted for clarity. Only B, O and S atoms are labelled.

**Figure 6:**
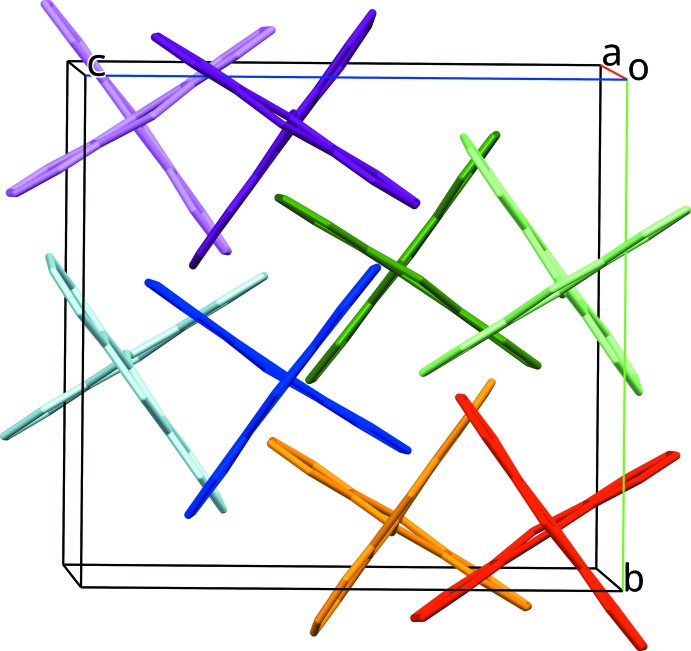
Crystal packing of (II). Mol­ecules occupying similar positions in (I) and (II) are drawn with the same colour to illustrate the similarities between (I) and (II).

**Table 1:** Comparison of B—C and S—S bond lengths and C—B—C and C—S—C bond angles (Å, °)

	B—C	C—B—C	S—C	C—S—C
LIPFAS	1.533	119.6	1.745	105.9
LIPFEW	1.546	119.4	1.745	106.5
QONKAG	1.534	119.6	1.740	106.2
(I)	1.539	121.8	1.758	106.4
(II)	1.548	120.2	1.746	106.3

Mean values of bond lengths [Å] and angles [°] in I and II and three structures (REFCODES given) from the CSD.

**Table 2:** Experimental details

	(I)	(II)
Crystal data		
Chemical formula	C_24_H_16_B_2_OS_2_	C_24_H_16_B_2_OS_2_
*M*_r_	406.11	406.11
Crystal system, space group	Monoclinic, *P*2_1_	Monoclinic, *P*2_1_/*c*
Temperature (K)	173	173
*a*, *b*, *c* (Å)	13.2989 (11), 12.7097 (7), 23.3300 (16)	23.2471 (18), 12.5949 (16), 13.3561 (11)
β (°)	93.727 (6)	92.217 (6)
*V* (Å^3^)	3935.0 (5)	3907.7 (7)
*Z*	8	8
Radiation type	Mo *K*α	Mo *K*α
μ (mm^−1^)	0.28	0.29
Crystal size (mm)	0.26 × 0.25 × 0.25	0.19 × 0.12 × 0.09

Data collection		
Diffractometer	Stoe *IPDS* II two-circle	Stoe *IPDS* II two-circle
Absorption correction	Multi-scan (*X-AREA*; Stoe & Cie, 2001 ▸)	Multi-scan (*X-AREA*; Stoe & Cie, 2001 ▸)
*T*_min_, *T*_max_	0.787, 1.000	0.677, 1.000
No. of measured, independent and observed [*I* > 2σ(*I*)] reflections	48969, 48969, 39838	32689, 32689, 13195
(sin θ/λ)_max_ (Å^−1^)	0.615	0.595

Refinement		
*R*[*F* ^2^ > 2σ(*F* ^2^)], *wR*(*F* ^2^), *S*	0.045, 0.082, 0.90	0.079, 0.184, 0.86
No. of reflections	48969	32689
No. of parameters	1046	524
No. of restraints	1	0
H-atom treatment	H-atom parameters constrained	H-atom parameters constrained
Δρ_max_, Δρ_min_ (e Å^−3^)	0.32, −0.25	0.48, −0.28
Absolute structure	Flack *x* determined using 5084 quotients [(*I* ^+^)−(*I* ^−^)]/[(*I* ^+^)+(*I* ^−^)] (Parsons *et al.*, 2013 ▸)	–
Absolute structure parameter	0.28 (3)	–

Computer programs: *X-AREA* (Stoe & Cie, 2001 ▸), *SHELXS97* and *XP* in *SHELXTL-Plus* (Sheldrick, 2008 ▸), *Mercury* (Macrae *et al.*, 2008 ▸), *SHELXL2014* (Sheldrick, 2015 ▸) and *publCIF* (Westrip, 2010 ▸).

**Table 3:** Systematic absence exceptions for (I) (space group *P*2_1_)

	-21-	-a-	-c-	-n-
N	54	2011	1973	1994
N[*I*>3σ(*I*)]	0	1185	1270	1219
mean intensity	0.3	2.8	5.6	5.6
mean *I*/σ(*I*)	1.0	7.4	11.6	11.5

*N*: number of reflection per group; *N*[*I*>3σ(*I*)]: number of reflections with *I* greater than 3σ(*I*).

**Table 4:** Systematic absence exceptions for (II) (space group *P*2_1_/*c*)

	-21-	-a-	-c-	-n-
*N*	14	1687	1697	1694
*N*(*I*>3σ(*I*))	0	330	103	350
mean intensity	0.3	5.0	0.7	5.1
mean *I*/σ(*I*)	0.9	5.0	1.5	5.1

*N*: number of reflection per group; *N*[*I*>3σ(*I*)]: number of reflections with *I* greater than 3σ(*I*).

## supplementary crystallographic information

### 10,10'-Oxybis(9-thia-10-hydro-10-boraanthracene) (I) Crystal data

**Table:**

C_24_H_16_B_2_OS_2_	*F*(000) = 1680
*M**_r_* = 406.11	*D*_x_ = 1.371 Mg m^−^^3^
Monoclinic, *P*2_1_	Mo *K*α radiation, λ = 0.71073 Å
*a* = 13.2989 (11) Å	Cell parameters from 16197 reflections
*b* = 12.7097 (7) Å	θ = 3.3–25.9°
*c* = 23.3300 (16) Å	µ = 0.28 mm^−^^1^
β = 93.727 (6)°	*T* = 173 K
*V* = 3935.0 (5) Å^3^	Block, colourless
*Z* = 8	0.26 × 0.25 × 0.25 mm

### 10,10'-Oxybis(9-thia-10-hydro-10-boraanthracene) (I) Data collection

**Table:**

Stoe IPDS II two-circle diffractometer	48969 independent reflections
Radiation source: Genix 3D IµS microfocus X-ray source	39838 reflections with *I* > 2σ(*I*)
ω scans	θ_max_ = 25.9°, θ_min_ = 3.4°
Absorption correction: multi-scan (X-Area; Stoe & Cie, 2001)	*h* = −16→16
*T*_min_ = 0.787, *T*_max_ = 1.000	*k* = −15→15
48969 measured reflections	*l* = −28→28

### 10,10'-Oxybis(9-thia-10-hydro-10-boraanthracene) (I) Refinement

**Table:**

Refinement on *F*^2^	Hydrogen site location: inferred from neighbouring sites
Least-squares matrix: full	H-atom parameters constrained
*R*[*F*^2^ > 2σ(*F*^2^)] = 0.045	*w* = 1/[σ^2^(*F*_o_^2^) + (0.0272*P*)^2^] where *P* = (*F*_o_^2^ + 2*F*_c_^2^)/3
*wR*(*F*^2^) = 0.082	(Δ/σ)_max_ = 0.001
*S* = 0.90	Δρ_max_ = 0.32 e Å^−^^3^
48969 reflections	Δρ_min_ = −0.25 e Å^−^^3^
1046 parameters	Absolute structure: Flack *x* determined using 5084 quotients [(*I*^+^)-(*I*^-^)]/[(*I*^+^)+(*I*^-^)] (Parsons *et al.*, 2013)
1 restraint	Absolute structure parameter: 0.28 (3)
Primary atom site location: structure-invariant direct methods	

### 10,10'-Oxybis(9-thia-10-hydro-10-boraanthracene) (I) Special details

**Table:**

Geometry. All esds (except the esd in the dihedral angle between two l.s. planes) are estimated using the full covariance matrix. The cell esds are taken into account individually in the estimation of esds in distances, angles and torsion angles; correlations between esds in cell parameters are only used when they are defined by crystal symmetry. An approximate (isotropic) treatment of cell esds is used for estimating esds involving l.s. planes.
Refinement. Refined as a 2-component twin.

### 10,10'-Oxybis(9-thia-10-hydro-10-boraanthracene) (I) Fractional atomic coordinates and isotropic or equivalent isotropic displacement parameters (Å^2^)

**Table:**

	*x*	*y*	*z*	*U*_iso_*/*U*_eq_	
S1A	0.89147 (14)	0.21177 (14)	−0.06026 (6)	0.0298 (4)	
S2A	0.90496 (15)	0.13029 (17)	0.31658 (7)	0.0455 (5)	
O1A	0.8426 (3)	0.2439 (4)	0.13221 (17)	0.0336 (11)	
B1A	0.8609 (6)	0.2311 (6)	0.0747 (3)	0.0265 (17)	
B2A	0.8614 (6)	0.2077 (7)	0.1862 (3)	0.0274 (17)	
C1A	0.9542 (5)	0.2799 (5)	0.0503 (3)	0.0257 (15)	
C2A	0.9708 (5)	0.2760 (5)	−0.0082 (3)	0.0252 (15)	
C3A	1.0560 (5)	0.3245 (5)	−0.0302 (3)	0.0312 (16)	
H3A	1.0654	0.3228	−0.0702	0.037*	
C4A	1.1257 (6)	0.3745 (6)	0.0075 (3)	0.0401 (19)	
H4A	1.1838	0.4062	−0.0068	0.048*	
C5A	1.1114 (6)	0.3785 (6)	0.0656 (3)	0.0429 (19)	
H5A	1.1590	0.4133	0.0911	0.052*	
C6A	1.0284 (6)	0.3320 (6)	0.0861 (3)	0.0366 (18)	
H6A	1.0201	0.3347	0.1262	0.044*	
C11A	0.7812 (5)	0.1704 (5)	0.0356 (3)	0.0258 (15)	
C12A	0.7920 (5)	0.1584 (5)	−0.0237 (3)	0.0242 (15)	
C13A	0.7216 (5)	0.1003 (5)	−0.0577 (3)	0.0307 (16)	
H13A	0.7299	0.0928	−0.0976	0.037*	
C14A	0.6403 (6)	0.0536 (6)	−0.0341 (3)	0.0333 (17)	
H14A	0.5934	0.0137	−0.0577	0.040*	
C15A	0.6268 (6)	0.0650 (6)	0.0244 (3)	0.0399 (19)	
H15A	0.5707	0.0337	0.0410	0.048*	
C16A	0.6963 (6)	0.1224 (6)	0.0575 (3)	0.0349 (17)	
H16A	0.6866	0.1300	0.0973	0.042*	
C21A	0.8145 (5)	0.2684 (6)	0.2350 (3)	0.0291 (16)	
C22A	0.8274 (5)	0.2385 (6)	0.2936 (3)	0.0341 (17)	
C23A	0.7834 (6)	0.2916 (7)	0.3374 (3)	0.048 (2)	
H23A	0.7915	0.2669	0.3759	0.058*	
C24A	0.7280 (6)	0.3800 (7)	0.3245 (3)	0.050 (2)	
H24A	0.6995	0.4186	0.3543	0.059*	
C25A	0.7132 (6)	0.4136 (7)	0.2684 (3)	0.048 (2)	
H25A	0.6739	0.4746	0.2598	0.058*	
C26A	0.7551 (6)	0.3594 (6)	0.2245 (3)	0.0405 (19)	
H26A	0.7436	0.3840	0.1862	0.049*	
C31A	0.9257 (5)	0.1074 (6)	0.1989 (3)	0.0317 (17)	
C32A	0.9480 (5)	0.0728 (6)	0.2551 (3)	0.0385 (18)	
C33A	1.0096 (6)	−0.0163 (6)	0.2661 (3)	0.047 (2)	
H33A	1.0246	−0.0390	0.3045	0.057*	
C34A	1.0479 (6)	−0.0704 (6)	0.2214 (4)	0.048 (2)	
H34A	1.0897	−0.1301	0.2290	0.058*	
C35A	1.0260 (5)	−0.0385 (6)	0.1655 (3)	0.0402 (17)	
H35A	1.0517	−0.0765	0.1346	0.048*	
C36A	0.9669 (5)	0.0486 (5)	0.1548 (3)	0.0331 (16)	
H36A	0.9531	0.0702	0.1161	0.040*	
S1B	0.88729 (15)	0.68772 (15)	0.17437 (7)	0.0365 (4)	
S2B	0.89332 (14)	0.73517 (14)	0.56106 (6)	0.0332 (4)	
O1B	0.8427 (4)	0.7147 (5)	0.36589 (19)	0.0488 (14)	
B1B	0.8557 (6)	0.7069 (7)	0.3093 (3)	0.034 (2)	
B2B	0.8564 (6)	0.7241 (6)	0.4239 (3)	0.0273 (18)	
C1B	0.8059 (5)	0.7855 (5)	0.2684 (3)	0.0302 (16)	
C2B	0.8154 (5)	0.7830 (6)	0.2089 (3)	0.0280 (15)	
C3B	0.7685 (5)	0.8556 (6)	0.1712 (3)	0.0380 (18)	
H3B	0.7769	0.8516	0.1312	0.046*	
C4B	0.7088 (6)	0.9345 (6)	0.1934 (3)	0.050 (2)	
H4B	0.6758	0.9841	0.1682	0.060*	
C5B	0.6977 (6)	0.9405 (6)	0.2510 (4)	0.048 (2)	
H5B	0.6572	0.9943	0.2659	0.057*	
C6B	0.7454 (5)	0.8684 (6)	0.2878 (3)	0.0379 (18)	
H6B	0.7373	0.8746	0.3278	0.046*	
C11B	0.9194 (5)	0.6136 (6)	0.2870 (3)	0.0307 (16)	
C12B	0.9361 (5)	0.6028 (6)	0.2288 (3)	0.0299 (16)	
C13B	0.9929 (6)	0.5182 (6)	0.2077 (3)	0.042 (2)	
H13B	1.0017	0.5115	0.1678	0.050*	
C14B	1.0352 (6)	0.4457 (6)	0.2466 (4)	0.047 (2)	
H14B	1.0749	0.3899	0.2332	0.056*	
C15B	1.0206 (6)	0.4534 (7)	0.3044 (3)	0.049 (2)	
H15B	1.0487	0.4022	0.3305	0.059*	
C16B	0.9645 (6)	0.5363 (6)	0.3242 (3)	0.042 (2)	
H16B	0.9560	0.5415	0.3642	0.050*	
C21B	0.9479 (5)	0.7871 (5)	0.4509 (3)	0.0312 (16)	
C22B	0.9658 (6)	0.7958 (5)	0.5104 (3)	0.0325 (17)	
C23B	1.0491 (6)	0.8539 (6)	0.5348 (3)	0.0416 (19)	
H23B	1.0604	0.8593	0.5753	0.050*	
C24B	1.1135 (7)	0.9023 (7)	0.4989 (4)	0.054 (2)	
H24B	1.1697	0.9410	0.5149	0.065*	
C25B	1.0967 (7)	0.8947 (7)	0.4397 (4)	0.052 (2)	
H25B	1.1415	0.9284	0.4154	0.062*	
C26B	1.0157 (6)	0.8390 (6)	0.4160 (3)	0.0418 (19)	
H26B	1.0051	0.8352	0.3754	0.050*	
C31B	0.7808 (5)	0.6710 (5)	0.4614 (3)	0.0273 (15)	
C32B	0.7926 (5)	0.6726 (5)	0.5221 (3)	0.0256 (15)	
C33B	0.7230 (6)	0.6218 (6)	0.5553 (3)	0.0355 (17)	
H33B	0.7322	0.6233	0.5960	0.043*	
C34B	0.6418 (6)	0.5701 (6)	0.5294 (3)	0.043 (2)	
H34B	0.5952	0.5354	0.5522	0.051*	
C35B	0.6275 (6)	0.5686 (6)	0.4695 (4)	0.044 (2)	
H35B	0.5708	0.5335	0.4516	0.052*	
C36B	0.6952 (6)	0.6177 (6)	0.4365 (3)	0.0388 (18)	
H36B	0.6843	0.6160	0.3959	0.047*	
S1C	0.61092 (14)	−0.08919 (13)	0.43175 (7)	0.0313 (4)	
S2C	0.61166 (15)	−0.07447 (15)	0.82018 (7)	0.0362 (4)	
O1C	0.6394 (4)	−0.0556 (4)	0.62671 (18)	0.0512 (15)	
B1C	0.6362 (7)	−0.0753 (6)	0.5685 (3)	0.033 (2)	
B2C	0.6316 (7)	−0.0595 (7)	0.6850 (3)	0.0325 (19)	
C1C	0.7171 (5)	−0.0266 (5)	0.5336 (3)	0.0275 (15)	
C2C	0.7105 (5)	−0.0304 (5)	0.4736 (3)	0.0271 (15)	
C3C	0.7844 (6)	0.0170 (6)	0.4420 (3)	0.0341 (17)	
H3C	0.7787	0.0148	0.4012	0.041*	
C4C	0.8645 (6)	0.0663 (6)	0.4698 (4)	0.044 (2)	
H4C	0.9142	0.0985	0.4481	0.053*	
C5C	0.8743 (6)	0.0702 (7)	0.5290 (4)	0.046 (2)	
H5C	0.9307	0.1041	0.5481	0.056*	
C6C	0.8022 (6)	0.0247 (6)	0.5602 (3)	0.0398 (19)	
H6C	0.8096	0.0276	0.6009	0.048*	
C11C	0.5500 (5)	−0.1443 (5)	0.5403 (3)	0.0318 (17)	
C12C	0.5356 (5)	−0.1519 (5)	0.4804 (3)	0.0312 (17)	
C13C	0.4552 (6)	−0.2107 (6)	0.4545 (4)	0.046 (2)	
H13C	0.4459	−0.2153	0.4138	0.055*	
C14C	0.3899 (6)	−0.2619 (7)	0.4892 (4)	0.058 (3)	
H14C	0.3350	−0.3008	0.4720	0.070*	
C15C	0.4034 (7)	−0.2571 (7)	0.5487 (4)	0.061 (3)	
H15C	0.3598	−0.2944	0.5721	0.073*	
C16C	0.4809 (7)	−0.1974 (6)	0.5730 (3)	0.043 (2)	
H16C	0.4882	−0.1918	0.6137	0.052*	
C21C	0.5655 (5)	0.0208 (5)	0.7130 (3)	0.0269 (15)	
C22C	0.5540 (5)	0.0191 (6)	0.7728 (3)	0.0268 (15)	
C23C	0.4943 (6)	0.0924 (6)	0.7988 (3)	0.0392 (19)	
H23C	0.4863	0.0885	0.8389	0.047*	
C24C	0.4466 (6)	0.1711 (6)	0.7663 (3)	0.0421 (19)	
H24C	0.4063	0.2216	0.7841	0.051*	
C25C	0.4575 (6)	0.1763 (7)	0.7077 (3)	0.046 (2)	
H25C	0.4243	0.2297	0.6852	0.056*	
C26C	0.5166 (6)	0.1037 (6)	0.6825 (3)	0.0379 (19)	
H26C	0.5249	0.1096	0.6425	0.045*	
C31C	0.6902 (5)	−0.1460 (5)	0.7193 (3)	0.0310 (16)	
C32C	0.6835 (6)	−0.1562 (6)	0.7788 (3)	0.0326 (17)	
C33C	0.7360 (6)	−0.2369 (6)	0.8093 (3)	0.0399 (18)	
H33C	0.7299	−0.2439	0.8495	0.048*	
C34C	0.7967 (6)	−0.3063 (6)	0.7813 (3)	0.0452 (19)	
H34C	0.8324	−0.3602	0.8023	0.054*	
C35C	0.8051 (5)	−0.2969 (6)	0.7224 (3)	0.0433 (19)	
H35C	0.8463	−0.3442	0.7029	0.052*	
C36C	0.7533 (5)	−0.2187 (6)	0.6925 (3)	0.0387 (18)	
H36C	0.7600	−0.2130	0.6524	0.046*	
S1D	0.63335 (16)	0.46013 (16)	0.67962 (7)	0.0452 (5)	
S2D	0.61249 (14)	0.39294 (14)	1.06599 (7)	0.0303 (4)	
O1D	0.6197 (4)	0.3876 (4)	0.87045 (18)	0.0462 (14)	
B1D	0.6239 (6)	0.4121 (7)	0.8135 (3)	0.034 (2)	
B2D	0.6190 (7)	0.3891 (6)	0.9298 (3)	0.033 (2)	
C1D	0.5722 (5)	0.5107 (6)	0.7887 (3)	0.0312 (16)	
C2D	0.5717 (6)	0.5347 (6)	0.7307 (3)	0.0344 (17)	
C3D	0.5199 (6)	0.6228 (6)	0.7067 (3)	0.0444 (19)	
H3D	0.5200	0.6366	0.6667	0.053*	
C4D	0.4701 (6)	0.6873 (7)	0.7412 (4)	0.054 (2)	
H4D	0.4349	0.7465	0.7251	0.065*	
C5D	0.4695 (6)	0.6687 (7)	0.7998 (4)	0.049 (2)	
H5D	0.4349	0.7150	0.8236	0.059*	
C6D	0.5197 (6)	0.5820 (6)	0.8230 (3)	0.043 (2)	
H6D	0.5193	0.5697	0.8632	0.051*	
C11D	0.6837 (5)	0.3374 (6)	0.7754 (3)	0.0329 (17)	
C12D	0.6906 (5)	0.3549 (5)	0.7170 (3)	0.0316 (16)	
C13D	0.7470 (6)	0.2852 (6)	0.6844 (3)	0.048 (2)	
H13D	0.7536	0.2982	0.6447	0.057*	
C14D	0.7923 (6)	0.1980 (8)	0.7104 (5)	0.062 (3)	
H14D	0.8292	0.1506	0.6883	0.075*	
C15D	0.7844 (6)	0.1798 (7)	0.7673 (4)	0.065 (3)	
H15D	0.8160	0.1196	0.7847	0.078*	
C16D	0.7318 (6)	0.2466 (6)	0.7997 (4)	0.050 (2)	
H16D	0.7272	0.2322	0.8394	0.060*	
C21D	0.7042 (5)	0.4460 (5)	0.9650 (3)	0.0276 (15)	
C22D	0.7055 (5)	0.4490 (5)	1.0250 (3)	0.0275 (15)	
C23D	0.7831 (5)	0.5002 (6)	1.0585 (3)	0.0340 (17)	
H23D	0.7823	0.5019	1.0992	0.041*	
C24D	0.8602 (6)	0.5480 (6)	1.0311 (3)	0.042 (2)	
H24D	0.9141	0.5806	1.0532	0.051*	
C25D	0.8598 (6)	0.5488 (6)	0.9715 (4)	0.045 (2)	
H25D	0.9119	0.5838	0.9531	0.054*	
C26D	0.7835 (6)	0.4986 (6)	0.9391 (3)	0.0370 (18)	
H26D	0.7843	0.4993	0.8984	0.044*	
C31D	0.5335 (5)	0.3302 (5)	0.9571 (3)	0.0244 (14)	
C32D	0.5271 (5)	0.3300 (5)	1.0169 (3)	0.0276 (15)	
C33D	0.4481 (6)	0.2760 (6)	1.0413 (3)	0.0365 (18)	
H33D	0.4449	0.2751	1.0818	0.044*	
C34D	0.3754 (6)	0.2244 (6)	1.0073 (3)	0.0414 (19)	
H34D	0.3224	0.1882	1.0244	0.050*	
C35D	0.3796 (6)	0.2255 (7)	0.9479 (3)	0.046 (2)	
H35D	0.3294	0.1906	0.9240	0.055*	
C36D	0.4572 (6)	0.2774 (6)	0.9242 (3)	0.0390 (18)	
H36D	0.4593	0.2777	0.8836	0.047*	

### 10,10'-Oxybis(9-thia-10-hydro-10-boraanthracene) (I) Atomic displacement parameters (Å^2^)

**Table:**

	*U*^11^	*U*^22^	*U*^33^	*U*^12^	*U*^13^	*U*^23^
S1A	0.0306 (10)	0.0384 (11)	0.0207 (7)	−0.0028 (8)	0.0032 (7)	0.0001 (7)
S2A	0.0469 (12)	0.0640 (13)	0.0254 (8)	−0.0038 (11)	0.0011 (8)	0.0113 (8)
O1A	0.037 (3)	0.040 (3)	0.024 (2)	0.005 (2)	0.0031 (19)	−0.002 (2)
B1A	0.035 (4)	0.020 (4)	0.025 (3)	0.011 (4)	0.002 (3)	0.006 (3)
B2A	0.021 (4)	0.032 (4)	0.030 (4)	−0.010 (4)	0.004 (3)	0.001 (3)
C1A	0.032 (4)	0.023 (4)	0.022 (3)	0.003 (3)	0.002 (3)	−0.004 (3)
C2A	0.020 (4)	0.024 (4)	0.031 (3)	0.003 (3)	−0.001 (3)	0.001 (3)
C3A	0.030 (4)	0.030 (4)	0.034 (3)	0.000 (3)	0.002 (3)	−0.003 (3)
C4A	0.028 (4)	0.038 (4)	0.055 (4)	−0.004 (3)	0.007 (3)	0.002 (4)
C5A	0.037 (4)	0.041 (5)	0.049 (4)	−0.016 (4)	−0.004 (4)	−0.007 (3)
C6A	0.054 (5)	0.032 (4)	0.024 (3)	0.000 (4)	0.001 (3)	−0.006 (3)
C11A	0.030 (4)	0.022 (4)	0.026 (3)	0.004 (3)	0.007 (3)	0.003 (3)
C12A	0.023 (4)	0.022 (4)	0.028 (3)	0.003 (3)	0.002 (3)	0.003 (3)
C13A	0.038 (4)	0.029 (4)	0.025 (3)	0.005 (3)	0.000 (3)	0.003 (3)
C14A	0.028 (4)	0.025 (4)	0.046 (4)	−0.001 (3)	0.001 (3)	−0.002 (3)
C15A	0.035 (4)	0.031 (4)	0.056 (5)	−0.007 (4)	0.019 (4)	−0.003 (4)
C16A	0.043 (4)	0.031 (4)	0.033 (3)	0.004 (4)	0.018 (3)	0.002 (3)
C21A	0.020 (3)	0.034 (4)	0.033 (3)	−0.005 (3)	0.003 (3)	−0.001 (3)
C22A	0.027 (4)	0.045 (4)	0.031 (3)	−0.010 (4)	0.006 (3)	−0.002 (3)
C23A	0.059 (5)	0.061 (6)	0.026 (3)	−0.021 (5)	0.013 (3)	−0.005 (3)
C24A	0.054 (5)	0.049 (5)	0.048 (4)	−0.017 (4)	0.022 (4)	−0.022 (4)
C25A	0.039 (5)	0.044 (5)	0.064 (5)	0.001 (4)	0.015 (4)	−0.012 (4)
C26A	0.042 (4)	0.050 (5)	0.030 (3)	−0.010 (4)	0.005 (3)	0.001 (3)
C31A	0.030 (4)	0.040 (4)	0.026 (3)	−0.010 (3)	0.005 (3)	0.003 (3)
C32A	0.030 (4)	0.039 (4)	0.046 (4)	−0.012 (4)	−0.004 (3)	0.011 (3)
C33A	0.038 (5)	0.046 (5)	0.057 (5)	−0.001 (4)	−0.004 (4)	0.025 (4)
C34A	0.029 (4)	0.031 (4)	0.084 (6)	0.000 (3)	0.004 (4)	0.013 (4)
C35A	0.025 (4)	0.033 (4)	0.063 (5)	−0.004 (3)	0.007 (3)	0.005 (4)
C36A	0.026 (4)	0.032 (4)	0.042 (4)	−0.006 (3)	0.004 (3)	0.008 (3)
S1B	0.0484 (12)	0.0391 (10)	0.0226 (7)	0.0079 (10)	0.0063 (8)	0.0007 (7)
S2B	0.0355 (10)	0.0422 (11)	0.0218 (7)	−0.0020 (9)	0.0019 (7)	−0.0033 (7)
O1B	0.052 (3)	0.069 (4)	0.026 (2)	−0.008 (3)	0.005 (2)	−0.003 (2)
B1B	0.028 (4)	0.045 (5)	0.027 (4)	−0.015 (4)	−0.001 (3)	−0.006 (4)
B2B	0.030 (4)	0.028 (4)	0.024 (3)	0.007 (4)	0.007 (3)	0.000 (3)
C1B	0.025 (4)	0.032 (4)	0.034 (3)	−0.009 (3)	0.007 (3)	−0.005 (3)
C2B	0.026 (4)	0.031 (4)	0.028 (3)	−0.008 (3)	0.005 (3)	0.001 (3)
C3B	0.029 (4)	0.047 (5)	0.039 (4)	0.001 (4)	0.007 (3)	0.011 (3)
C4B	0.040 (5)	0.044 (5)	0.067 (5)	0.003 (4)	0.013 (4)	0.016 (4)
C5B	0.031 (4)	0.030 (5)	0.084 (6)	0.001 (3)	0.019 (4)	−0.003 (4)
C6B	0.036 (4)	0.044 (5)	0.035 (4)	−0.011 (4)	0.011 (3)	−0.014 (3)
C11B	0.029 (4)	0.036 (4)	0.027 (3)	−0.003 (3)	−0.005 (3)	0.007 (3)
C12B	0.024 (4)	0.032 (4)	0.034 (4)	−0.003 (3)	0.001 (3)	0.000 (3)
C13B	0.043 (5)	0.041 (5)	0.042 (4)	−0.004 (4)	0.004 (4)	−0.002 (4)
C14B	0.039 (5)	0.031 (5)	0.070 (5)	0.002 (4)	−0.001 (4)	0.001 (4)
C15B	0.048 (5)	0.040 (5)	0.058 (5)	−0.003 (4)	−0.011 (4)	0.018 (4)
C16B	0.046 (5)	0.047 (5)	0.032 (4)	−0.013 (4)	−0.003 (3)	0.007 (3)
C21B	0.037 (4)	0.027 (4)	0.031 (3)	0.004 (3)	0.006 (3)	0.006 (3)
C22B	0.032 (4)	0.026 (4)	0.040 (4)	0.006 (3)	0.006 (3)	−0.003 (3)
C23B	0.033 (4)	0.047 (5)	0.043 (4)	−0.003 (4)	−0.006 (3)	−0.015 (4)
C24B	0.038 (5)	0.041 (5)	0.084 (6)	−0.013 (4)	0.007 (4)	−0.012 (4)
C25B	0.046 (5)	0.048 (5)	0.062 (5)	−0.008 (5)	0.013 (4)	0.005 (4)
C26B	0.044 (5)	0.043 (5)	0.040 (4)	−0.003 (4)	0.011 (4)	0.007 (3)
C31B	0.034 (4)	0.022 (4)	0.026 (3)	0.002 (3)	−0.001 (3)	−0.001 (3)
C32B	0.026 (4)	0.023 (4)	0.028 (3)	−0.001 (3)	0.005 (3)	−0.003 (3)
C33B	0.043 (4)	0.035 (4)	0.029 (3)	0.000 (4)	0.008 (3)	0.000 (3)
C34B	0.048 (5)	0.031 (5)	0.050 (5)	−0.005 (4)	0.016 (4)	0.008 (3)
C35B	0.039 (5)	0.032 (4)	0.060 (5)	−0.007 (4)	−0.002 (4)	0.002 (4)
C36B	0.042 (4)	0.037 (4)	0.037 (4)	0.000 (4)	−0.003 (4)	0.002 (3)
S1C	0.0368 (10)	0.0352 (10)	0.0222 (7)	−0.0077 (8)	0.0049 (7)	−0.0031 (7)
S2C	0.0479 (12)	0.0405 (11)	0.0206 (7)	0.0075 (9)	0.0053 (8)	0.0008 (7)
O1C	0.072 (4)	0.059 (4)	0.024 (2)	0.010 (3)	0.010 (2)	0.001 (2)
B1C	0.049 (5)	0.035 (5)	0.017 (3)	0.014 (4)	0.013 (3)	0.004 (3)
B2C	0.036 (5)	0.040 (5)	0.021 (3)	−0.007 (4)	0.003 (3)	−0.005 (3)
C1C	0.029 (4)	0.027 (4)	0.027 (3)	0.003 (3)	0.001 (3)	0.000 (3)
C2C	0.029 (4)	0.024 (4)	0.028 (3)	0.001 (3)	0.002 (3)	−0.003 (3)
C3C	0.038 (4)	0.029 (4)	0.035 (4)	−0.003 (3)	0.007 (3)	−0.003 (3)
C4C	0.038 (5)	0.036 (5)	0.058 (5)	−0.009 (4)	0.002 (4)	0.006 (4)
C5C	0.035 (5)	0.041 (5)	0.062 (5)	−0.016 (4)	−0.012 (4)	−0.002 (4)
C6C	0.045 (5)	0.041 (5)	0.031 (4)	0.002 (4)	−0.012 (3)	−0.005 (3)
C11C	0.036 (4)	0.021 (4)	0.040 (4)	0.009 (3)	0.018 (3)	0.010 (3)
C12C	0.031 (4)	0.025 (4)	0.038 (4)	0.002 (3)	0.005 (3)	−0.001 (3)
C13C	0.042 (5)	0.035 (4)	0.060 (5)	−0.005 (4)	0.007 (4)	−0.007 (4)
C14C	0.034 (5)	0.032 (5)	0.109 (7)	−0.012 (4)	0.014 (5)	−0.011 (5)
C15C	0.056 (6)	0.035 (5)	0.096 (7)	0.003 (5)	0.039 (5)	0.006 (5)
C16C	0.051 (5)	0.032 (4)	0.050 (4)	0.005 (4)	0.024 (4)	0.011 (4)
C21C	0.020 (3)	0.032 (4)	0.029 (3)	0.000 (3)	0.003 (3)	0.000 (3)
C22C	0.021 (4)	0.033 (4)	0.026 (3)	0.002 (3)	−0.001 (3)	−0.003 (3)
C23C	0.043 (5)	0.043 (5)	0.031 (3)	0.007 (4)	0.001 (3)	−0.006 (3)
C24C	0.036 (4)	0.039 (4)	0.051 (4)	0.007 (4)	0.000 (3)	−0.011 (4)
C25C	0.046 (5)	0.037 (5)	0.055 (4)	0.009 (4)	−0.005 (4)	0.004 (4)
C26C	0.041 (4)	0.046 (5)	0.026 (3)	0.001 (4)	0.000 (3)	0.007 (3)
C31C	0.032 (4)	0.032 (4)	0.029 (3)	−0.003 (3)	0.007 (3)	−0.009 (3)
C32C	0.028 (4)	0.031 (4)	0.039 (4)	0.000 (3)	0.005 (3)	−0.003 (3)
C33C	0.038 (4)	0.045 (5)	0.036 (4)	0.001 (4)	0.003 (3)	0.004 (3)
C34C	0.033 (4)	0.038 (4)	0.064 (5)	0.008 (4)	−0.002 (4)	0.006 (4)
C35C	0.029 (4)	0.039 (5)	0.063 (5)	0.008 (4)	0.010 (4)	−0.009 (4)
C36C	0.035 (4)	0.041 (5)	0.040 (4)	−0.004 (4)	0.007 (3)	−0.012 (3)
S1D	0.0579 (13)	0.0541 (13)	0.0248 (8)	0.0059 (11)	0.0120 (8)	0.0061 (8)
S2D	0.0324 (10)	0.0351 (11)	0.0235 (8)	−0.0040 (8)	0.0041 (7)	0.0005 (7)
O1D	0.050 (3)	0.061 (3)	0.028 (2)	−0.010 (3)	0.004 (2)	0.009 (2)
B1D	0.034 (5)	0.051 (5)	0.016 (3)	−0.015 (4)	−0.003 (3)	−0.001 (3)
B2D	0.047 (5)	0.031 (5)	0.021 (3)	0.010 (4)	0.002 (4)	0.005 (3)
C1D	0.023 (4)	0.032 (4)	0.039 (4)	−0.003 (3)	0.008 (3)	0.000 (3)
C2D	0.035 (4)	0.032 (4)	0.037 (4)	−0.007 (3)	0.007 (3)	0.007 (3)
C3D	0.046 (5)	0.036 (4)	0.052 (4)	0.005 (4)	0.004 (4)	0.015 (4)
C4D	0.032 (4)	0.035 (5)	0.096 (6)	0.000 (4)	0.000 (4)	0.016 (5)
C5D	0.033 (4)	0.041 (5)	0.074 (5)	0.002 (4)	0.014 (4)	−0.013 (4)
C6D	0.038 (4)	0.045 (5)	0.047 (4)	−0.010 (4)	0.013 (4)	−0.007 (4)
C11D	0.023 (4)	0.030 (4)	0.045 (4)	−0.004 (3)	−0.005 (3)	0.010 (3)
C12D	0.024 (4)	0.033 (4)	0.038 (4)	−0.004 (3)	0.006 (3)	−0.003 (3)
C13D	0.045 (5)	0.050 (5)	0.050 (5)	−0.013 (4)	0.011 (4)	−0.017 (4)
C14D	0.035 (5)	0.043 (5)	0.110 (8)	0.007 (4)	0.013 (5)	−0.024 (6)
C15D	0.037 (5)	0.043 (5)	0.116 (8)	0.009 (4)	0.001 (5)	0.006 (6)
C16D	0.040 (4)	0.040 (5)	0.067 (5)	−0.002 (4)	−0.010 (4)	0.006 (4)
C21D	0.026 (4)	0.019 (3)	0.039 (3)	0.006 (3)	0.011 (3)	0.001 (3)
C22D	0.025 (4)	0.020 (4)	0.039 (4)	0.005 (3)	0.008 (3)	0.001 (3)
C23D	0.036 (4)	0.031 (4)	0.036 (4)	0.003 (3)	0.005 (3)	−0.002 (3)
C24D	0.036 (4)	0.035 (5)	0.056 (5)	0.000 (4)	0.008 (4)	−0.007 (4)
C25D	0.034 (4)	0.037 (5)	0.065 (5)	−0.008 (4)	0.019 (4)	0.000 (4)
C26D	0.044 (5)	0.032 (4)	0.037 (4)	−0.002 (4)	0.013 (3)	−0.001 (3)
C31D	0.024 (3)	0.019 (3)	0.031 (3)	0.001 (3)	0.000 (3)	0.005 (3)
C32D	0.028 (4)	0.015 (3)	0.040 (4)	0.002 (3)	0.005 (3)	−0.002 (3)
C33D	0.040 (4)	0.036 (4)	0.035 (4)	0.000 (4)	0.012 (3)	0.001 (3)
C34D	0.030 (4)	0.032 (4)	0.063 (4)	−0.007 (3)	0.011 (4)	0.002 (4)
C35D	0.039 (5)	0.042 (5)	0.055 (4)	−0.016 (4)	−0.003 (4)	−0.008 (4)
C36D	0.038 (4)	0.037 (4)	0.042 (4)	−0.006 (4)	−0.006 (4)	−0.001 (3)

### 10,10'-Oxybis(9-thia-10-hydro-10-boraanthracene) (I) Geometric parameters (Å, º)

**Table:**

S1A—C12A	1.756 (6)	S1C—C12C	1.753 (7)
S1A—C2A	1.757 (7)	S1C—C2C	1.760 (7)
S2A—C32A	1.739 (8)	S2C—C32C	1.745 (7)
S2A—C22A	1.781 (8)	S2C—C22C	1.765 (7)
O1A—B2A	1.349 (8)	O1C—B2C	1.372 (8)
O1A—B1A	1.388 (8)	O1C—B1C	1.378 (8)
B1A—C1A	1.530 (10)	B1C—C1C	1.523 (10)
B1A—C11A	1.557 (10)	B1C—C11C	1.555 (11)
B2A—C21A	1.542 (10)	B2C—C21C	1.521 (10)
B2A—C31A	1.552 (11)	B2C—C31C	1.540 (11)
C1A—C2A	1.397 (8)	C1C—C2C	1.397 (8)
C1A—C6A	1.416 (10)	C1C—C6C	1.415 (10)
C2A—C3A	1.414 (10)	C2C—C3C	1.403 (9)
C3A—C4A	1.390 (10)	C3C—C4C	1.363 (11)
C3A—H3A	0.9500	C3C—H3C	0.9500
C4A—C5A	1.382 (9)	C4C—C5C	1.382 (11)
C4A—H4A	0.9500	C4C—H4C	0.9500
C5A—C6A	1.366 (10)	C5C—C6C	1.368 (11)
C5A—H5A	0.9500	C5C—H5C	0.9500
C6A—H6A	0.9500	C6C—H6C	0.9500
C11A—C16A	1.407 (9)	C11C—C12C	1.401 (9)
C11A—C12A	1.411 (8)	C11C—C16C	1.406 (9)
C12A—C13A	1.397 (9)	C12C—C13C	1.409 (11)
C13A—C14A	1.379 (10)	C13C—C14C	1.387 (11)
C13A—H13A	0.9500	C13C—H13C	0.9500
C14A—C15A	1.395 (10)	C14C—C15C	1.389 (12)
C14A—H14A	0.9500	C14C—H14C	0.9500
C15A—C16A	1.376 (11)	C15C—C16C	1.372 (13)
C15A—H15A	0.9500	C15C—H15C	0.9500
C16A—H16A	0.9500	C16C—H16C	0.9500
C21A—C26A	1.413 (10)	C21C—C26C	1.408 (10)
C21A—C22A	1.417 (9)	C21C—C22C	1.412 (8)
C22A—C23A	1.387 (9)	C22C—C23C	1.389 (10)
C23A—C24A	1.366 (11)	C23C—C24C	1.384 (10)
C23A—H23A	0.9500	C23C—H23C	0.9500
C24A—C25A	1.378 (11)	C24C—C25C	1.386 (10)
C24A—H24A	0.9500	C24C—H24C	0.9500
C25A—C26A	1.380 (10)	C25C—C26C	1.368 (10)
C25A—H25A	0.9500	C25C—H25C	0.9500
C26A—H26A	0.9500	C26C—H26C	0.9500
C31A—C32A	1.398 (9)	C31C—C32C	1.403 (9)
C31A—C36A	1.410 (9)	C31C—C36C	1.419 (9)
C32A—C33A	1.411 (10)	C32C—C33C	1.409 (10)
C33A—C34A	1.375 (11)	C33C—C34C	1.387 (10)
C33A—H33A	0.9500	C33C—H33C	0.9500
C34A—C35A	1.377 (10)	C34C—C35C	1.391 (10)
C34A—H34A	0.9500	C34C—H34C	0.9500
C35A—C36A	1.371 (9)	C35C—C36C	1.373 (10)
C35A—H35A	0.9500	C35C—H35C	0.9500
C36A—H36A	0.9500	C36C—H36C	0.9500
S1B—C12B	1.758 (7)	S1D—C12D	1.744 (7)
S1B—C2B	1.770 (7)	S1D—C2D	1.765 (7)
S2B—C22B	1.752 (7)	S2D—C32D	1.753 (7)
S2B—C32B	1.759 (7)	S2D—C22D	1.763 (7)
O1B—B1B	1.346 (8)	O1D—B1D	1.370 (8)
O1B—B2B	1.358 (8)	O1D—B2D	1.386 (8)
B1B—C1B	1.506 (11)	B1D—C1D	1.525 (11)
B1B—C11B	1.567 (11)	B1D—C11D	1.553 (11)
B2B—C31B	1.534 (10)	B2D—C31D	1.533 (11)
B2B—C21B	1.555 (11)	B2D—C21D	1.536 (11)
C1B—C2B	1.401 (8)	C1D—C2D	1.387 (9)
C1B—C6B	1.418 (10)	C1D—C6D	1.422 (10)
C2B—C3B	1.394 (10)	C2D—C3D	1.410 (10)
C3B—C4B	1.399 (10)	C3D—C4D	1.352 (11)
C3B—H3B	0.9500	C3D—H3D	0.9500
C4B—C5B	1.364 (10)	C4D—C5D	1.387 (10)
C4B—H4B	0.9500	C4D—H4D	0.9500
C5B—C6B	1.381 (11)	C5D—C6D	1.380 (11)
C5B—H5B	0.9500	C5D—H5D	0.9500
C6B—H6B	0.9500	C6D—H6D	0.9500
C11B—C12B	1.397 (9)	C11D—C12D	1.391 (9)
C11B—C16B	1.418 (10)	C11D—C16D	1.419 (10)
C12B—C13B	1.419 (11)	C12D—C13D	1.414 (10)
C13B—C14B	1.386 (11)	C13D—C14D	1.383 (12)
C13B—H13B	0.9500	C13D—H13D	0.9500
C14B—C15B	1.379 (10)	C14D—C15D	1.358 (12)
C14B—H14B	0.9500	C14D—H14D	0.9500
C15B—C16B	1.388 (11)	C15D—C16D	1.360 (12)
C15B—H15B	0.9500	C15D—H15D	0.9500
C16B—H16B	0.9500	C16D—H16D	0.9500
C21B—C22B	1.398 (9)	C21D—C22D	1.398 (9)
C21B—C26B	1.416 (10)	C21D—C26D	1.416 (10)
C22B—C23B	1.418 (10)	C22D—C23D	1.413 (10)
C23B—C24B	1.380 (11)	C23D—C24D	1.385 (10)
C23B—H23B	0.9500	C23D—H23D	0.9500
C24B—C25B	1.387 (11)	C24D—C25D	1.388 (11)
C24B—H24B	0.9500	C24D—H24D	0.9500
C25B—C26B	1.375 (12)	C25D—C26D	1.381 (11)
C25B—H25B	0.9500	C25D—H25D	0.9500
C26B—H26B	0.9500	C26D—H26D	0.9500
C31B—C32B	1.415 (8)	C31D—C36D	1.403 (10)
C31B—C36B	1.416 (10)	C31D—C32D	1.403 (8)
C32B—C33B	1.402 (9)	C32D—C33D	1.407 (10)
C33B—C34B	1.371 (11)	C33D—C34D	1.376 (10)
C33B—H33B	0.9500	C33D—H33D	0.9500
C34B—C35B	1.398 (10)	C34D—C35D	1.391 (10)
C34B—H34B	0.9500	C34D—H34D	0.9500
C35B—C36B	1.372 (10)	C35D—C36D	1.371 (10)
C35B—H35B	0.9500	C35D—H35D	0.9500
C36B—H36B	0.9500	C36D—H36D	0.9500
			
C12A—S1A—C2A	106.4 (3)	C12C—S1C—C2C	105.9 (3)
C32A—S2A—C22A	106.9 (3)	C32C—S2C—C22C	106.6 (3)
B2A—O1A—B1A	146.0 (6)	B2C—O1C—B1C	166.0 (7)
O1A—B1A—C1A	120.7 (6)	O1C—B1C—C1C	118.4 (7)
O1A—B1A—C11A	117.9 (6)	O1C—B1C—C11C	119.7 (6)
C1A—B1A—C11A	121.4 (5)	C1C—B1C—C11C	121.9 (5)
O1A—B2A—C21A	117.4 (7)	O1C—B2C—C21C	119.0 (7)
O1A—B2A—C31A	121.7 (6)	O1C—B2C—C31C	118.1 (7)
C21A—B2A—C31A	121.0 (6)	C21C—B2C—C31C	122.9 (5)
C2A—C1A—C6A	116.4 (6)	C2C—C1C—C6C	116.8 (6)
C2A—C1A—B1A	122.2 (6)	C2C—C1C—B1C	121.5 (6)
C6A—C1A—B1A	121.3 (6)	C6C—C1C—B1C	121.7 (6)
C1A—C2A—C3A	121.3 (6)	C1C—C2C—C3C	120.7 (6)
C1A—C2A—S1A	124.4 (5)	C1C—C2C—S1C	124.5 (5)
C3A—C2A—S1A	114.3 (5)	C3C—C2C—S1C	114.8 (5)
C4A—C3A—C2A	119.2 (6)	C4C—C3C—C2C	120.1 (7)
C4A—C3A—H3A	120.4	C4C—C3C—H3C	120.0
C2A—C3A—H3A	120.4	C2C—C3C—H3C	120.0
C5A—C4A—C3A	120.5 (7)	C3C—C4C—C5C	120.8 (8)
C5A—C4A—H4A	119.7	C3C—C4C—H4C	119.6
C3A—C4A—H4A	119.7	C5C—C4C—H4C	119.6
C6A—C5A—C4A	119.6 (7)	C6C—C5C—C4C	119.5 (7)
C6A—C5A—H5A	120.2	C6C—C5C—H5C	120.2
C4A—C5A—H5A	120.2	C4C—C5C—H5C	120.2
C5A—C6A—C1A	122.9 (6)	C5C—C6C—C1C	122.1 (7)
C5A—C6A—H6A	118.5	C5C—C6C—H6C	119.0
C1A—C6A—H6A	118.5	C1C—C6C—H6C	119.0
C16A—C11A—C12A	116.4 (6)	C12C—C11C—C16C	117.2 (7)
C16A—C11A—B1A	122.3 (6)	C12C—C11C—B1C	120.7 (6)
C12A—C11A—B1A	121.3 (6)	C16C—C11C—B1C	122.0 (7)
C13A—C12A—C11A	120.4 (6)	C11C—C12C—C13C	121.0 (7)
C13A—C12A—S1A	115.3 (5)	C11C—C12C—S1C	124.6 (5)
C11A—C12A—S1A	124.3 (5)	C13C—C12C—S1C	114.4 (6)
C14A—C13A—C12A	121.0 (6)	C14C—C13C—C12C	119.0 (8)
C14A—C13A—H13A	119.5	C14C—C13C—H13C	120.5
C12A—C13A—H13A	119.5	C12C—C13C—H13C	120.5
C13A—C14A—C15A	119.9 (7)	C13C—C14C—C15C	121.2 (8)
C13A—C14A—H14A	120.0	C13C—C14C—H14C	119.4
C15A—C14A—H14A	120.0	C15C—C14C—H14C	119.4
C16A—C15A—C14A	118.7 (7)	C16C—C15C—C14C	118.9 (8)
C16A—C15A—H15A	120.6	C16C—C15C—H15C	120.6
C14A—C15A—H15A	120.6	C14C—C15C—H15C	120.6
C15A—C16A—C11A	123.5 (6)	C15C—C16C—C11C	122.7 (8)
C15A—C16A—H16A	118.3	C15C—C16C—H16C	118.7
C11A—C16A—H16A	118.3	C11C—C16C—H16C	118.7
C26A—C21A—C22A	114.7 (6)	C26C—C21C—C22C	115.8 (6)
C26A—C21A—B2A	122.0 (6)	C26C—C21C—B2C	123.1 (6)
C22A—C21A—B2A	123.3 (7)	C22C—C21C—B2C	121.1 (6)
C23A—C22A—C21A	123.3 (7)	C23C—C22C—C21C	121.5 (6)
C23A—C22A—S2A	114.7 (6)	C23C—C22C—S2C	114.6 (5)
C21A—C22A—S2A	122.0 (5)	C21C—C22C—S2C	123.9 (5)
C24A—C23A—C22A	119.0 (7)	C24C—C23C—C22C	120.0 (7)
C24A—C23A—H23A	120.5	C24C—C23C—H23C	120.0
C22A—C23A—H23A	120.5	C22C—C23C—H23C	120.0
C23A—C24A—C25A	120.4 (7)	C23C—C24C—C25C	120.1 (7)
C23A—C24A—H24A	119.8	C23C—C24C—H24C	120.0
C25A—C24A—H24A	119.8	C25C—C24C—H24C	120.0
C24A—C25A—C26A	120.7 (8)	C26C—C25C—C24C	119.4 (7)
C24A—C25A—H25A	119.7	C26C—C25C—H25C	120.3
C26A—C25A—H25A	119.7	C24C—C25C—H25C	120.3
C25A—C26A—C21A	121.8 (7)	C25C—C26C—C21C	123.1 (6)
C25A—C26A—H26A	119.1	C25C—C26C—H26C	118.4
C21A—C26A—H26A	119.1	C21C—C26C—H26C	118.4
C32A—C31A—C36A	116.6 (7)	C32C—C31C—C36C	116.9 (7)
C32A—C31A—B2A	121.2 (6)	C32C—C31C—B2C	121.1 (6)
C36A—C31A—B2A	122.1 (6)	C36C—C31C—B2C	122.0 (6)
C31A—C32A—C33A	120.6 (7)	C31C—C32C—C33C	120.5 (6)
C31A—C32A—S2A	125.5 (6)	C31C—C32C—S2C	124.4 (6)
C33A—C32A—S2A	114.0 (6)	C33C—C32C—S2C	115.1 (5)
C34A—C33A—C32A	120.2 (7)	C34C—C33C—C32C	120.6 (6)
C34A—C33A—H33A	119.9	C34C—C33C—H33C	119.7
C32A—C33A—H33A	119.9	C32C—C33C—H33C	119.7
C33A—C34A—C35A	120.3 (7)	C33C—C34C—C35C	119.8 (7)
C33A—C34A—H34A	119.9	C33C—C34C—H34C	120.1
C35A—C34A—H34A	119.9	C35C—C34C—H34C	120.1
C36A—C35A—C34A	119.6 (7)	C36C—C35C—C34C	119.6 (7)
C36A—C35A—H35A	120.2	C36C—C35C—H35C	120.2
C34A—C35A—H35A	120.2	C34C—C35C—H35C	120.2
C35A—C36A—C31A	122.7 (7)	C35C—C36C—C31C	122.7 (7)
C35A—C36A—H36A	118.6	C35C—C36C—H36C	118.7
C31A—C36A—H36A	118.6	C31C—C36C—H36C	118.7
C12B—S1B—C2B	106.0 (3)	C12D—S1D—C2D	106.4 (3)
C22B—S2B—C32B	106.5 (3)	C32D—S2D—C22D	106.1 (3)
B1B—O1B—B2B	164.8 (7)	B1D—O1D—B2D	165.9 (7)
O1B—B1B—C1B	119.6 (7)	O1D—B1D—C1D	120.6 (7)
O1B—B1B—C11B	119.3 (7)	O1D—B1D—C11D	118.0 (7)
C1B—B1B—C11B	121.1 (6)	C1D—B1D—C11D	121.4 (6)
O1B—B2B—C31B	118.6 (7)	O1D—B2D—C31D	117.4 (7)
O1B—B2B—C21B	120.0 (6)	O1D—B2D—C21D	119.2 (7)
C31B—B2B—C21B	121.3 (6)	C31D—B2D—C21D	123.3 (6)
C2B—C1B—C6B	115.0 (6)	C2D—C1D—C6D	116.0 (7)
C2B—C1B—B1B	123.2 (6)	C2D—C1D—B1D	121.6 (6)
C6B—C1B—B1B	121.7 (6)	C6D—C1D—B1D	122.4 (6)
C3B—C2B—C1B	123.0 (7)	C1D—C2D—C3D	122.1 (7)
C3B—C2B—S1B	113.5 (5)	C1D—C2D—S1D	124.6 (6)
C1B—C2B—S1B	123.6 (5)	C3D—C2D—S1D	113.3 (5)
C2B—C3B—C4B	118.8 (6)	C4D—C3D—C2D	119.4 (7)
C2B—C3B—H3B	120.6	C4D—C3D—H3D	120.3
C4B—C3B—H3B	120.6	C2D—C3D—H3D	120.3
C5B—C4B—C3B	120.3 (7)	C3D—C4D—C5D	121.1 (8)
C5B—C4B—H4B	119.8	C3D—C4D—H4D	119.5
C3B—C4B—H4B	119.8	C5D—C4D—H4D	119.5
C4B—C5B—C6B	120.1 (7)	C6D—C5D—C4D	119.4 (7)
C4B—C5B—H5B	120.0	C6D—C5D—H5D	120.3
C6B—C5B—H5B	120.0	C4D—C5D—H5D	120.3
C5B—C6B—C1B	122.8 (6)	C5D—C6D—C1D	122.0 (7)
C5B—C6B—H6B	118.6	C5D—C6D—H6D	119.0
C1B—C6B—H6B	118.6	C1D—C6D—H6D	119.0
C12B—C11B—C16B	116.1 (7)	C12D—C11D—C16D	117.6 (7)
C12B—C11B—B1B	121.4 (6)	C12D—C11D—B1D	122.1 (6)
C16B—C11B—B1B	122.5 (6)	C16D—C11D—B1D	120.3 (7)
C11B—C12B—C13B	122.1 (7)	C11D—C12D—C13D	119.9 (7)
C11B—C12B—S1B	124.6 (5)	C11D—C12D—S1D	123.9 (5)
C13B—C12B—S1B	113.3 (5)	C13D—C12D—S1D	116.2 (5)
C14B—C13B—C12B	118.7 (7)	C14D—C13D—C12D	119.8 (7)
C14B—C13B—H13B	120.6	C14D—C13D—H13D	120.1
C12B—C13B—H13B	120.6	C12D—C13D—H13D	120.1
C15B—C14B—C13B	121.1 (8)	C15D—C14D—C13D	120.5 (8)
C15B—C14B—H14B	119.5	C15D—C14D—H14D	119.8
C13B—C14B—H14B	119.5	C13D—C14D—H14D	119.8
C14B—C15B—C16B	119.5 (7)	C14D—C15D—C16D	120.7 (9)
C14B—C15B—H15B	120.3	C14D—C15D—H15D	119.6
C16B—C15B—H15B	120.3	C16D—C15D—H15D	119.6
C15B—C16B—C11B	122.5 (7)	C15D—C16D—C11D	121.4 (8)
C15B—C16B—H16B	118.7	C15D—C16D—H16D	119.3
C11B—C16B—H16B	118.7	C11D—C16D—H16D	119.3
C22B—C21B—C26B	117.4 (7)	C22D—C21D—C26D	116.9 (7)
C22B—C21B—B2B	121.4 (6)	C22D—C21D—B2D	120.5 (6)
C26B—C21B—B2B	121.2 (6)	C26D—C21D—B2D	122.6 (6)
C21B—C22B—C23B	121.2 (7)	C21D—C22D—C23D	121.9 (6)
C21B—C22B—S2B	124.7 (6)	C21D—C22D—S2D	124.7 (5)
C23B—C22B—S2B	114.1 (5)	C23D—C22D—S2D	113.5 (5)
C24B—C23B—C22B	119.2 (7)	C24D—C23D—C22D	118.8 (7)
C24B—C23B—H23B	120.4	C24D—C23D—H23D	120.6
C22B—C23B—H23B	120.4	C22D—C23D—H23D	120.6
C23B—C24B—C25B	120.4 (8)	C23D—C24D—C25D	120.8 (8)
C23B—C24B—H24B	119.8	C23D—C24D—H24D	119.6
C25B—C24B—H24B	119.8	C25D—C24D—H24D	119.6
C26B—C25B—C24B	120.5 (7)	C26D—C25D—C24D	120.0 (7)
C26B—C25B—H25B	119.8	C26D—C25D—H25D	120.0
C24B—C25B—H25B	119.8	C24D—C25D—H25D	120.0
C25B—C26B—C21B	121.3 (7)	C25D—C26D—C21D	121.7 (7)
C25B—C26B—H26B	119.3	C25D—C26D—H26D	119.2
C21B—C26B—H26B	119.3	C21D—C26D—H26D	119.2
C32B—C31B—C36B	116.9 (6)	C36D—C31D—C32D	117.0 (6)
C32B—C31B—B2B	122.1 (6)	C36D—C31D—B2D	122.5 (6)
C36B—C31B—B2B	121.0 (6)	C32D—C31D—B2D	120.5 (6)
C33B—C32B—C31B	120.8 (6)	C31D—C32D—C33D	119.9 (7)
C33B—C32B—S2B	115.5 (5)	C31D—C32D—S2D	124.8 (5)
C31B—C32B—S2B	123.7 (5)	C33D—C32D—S2D	115.3 (5)
C34B—C33B—C32B	120.5 (7)	C34D—C33D—C32D	121.0 (7)
C34B—C33B—H33B	119.8	C34D—C33D—H33D	119.5
C32B—C33B—H33B	119.8	C32D—C33D—H33D	119.5
C33B—C34B—C35B	119.9 (7)	C33D—C34D—C35D	119.8 (7)
C33B—C34B—H34B	120.0	C33D—C34D—H34D	120.1
C35B—C34B—H34B	120.0	C35D—C34D—H34D	120.1
C36B—C35B—C34B	120.2 (8)	C36D—C35D—C34D	119.1 (7)
C36B—C35B—H35B	119.9	C36D—C35D—H35D	120.5
C34B—C35B—H35B	119.9	C34D—C35D—H35D	120.5
C35B—C36B—C31B	121.7 (7)	C35D—C36D—C31D	123.2 (7)
C35B—C36B—H36B	119.1	C35D—C36D—H36D	118.4
C31B—C36B—H36B	119.1	C31D—C36D—H36D	118.4
			
B2A—O1A—B1A—C1A	−85.0 (12)	B2C—O1C—B1C—C1C	−151 (3)
B2A—O1A—B1A—C11A	97.8 (11)	B2C—O1C—B1C—C11C	30 (3)
B1A—O1A—B2A—C21A	177.5 (9)	B1C—O1C—B2C—C21C	−116 (3)
B1A—O1A—B2A—C31A	−3.8 (14)	B1C—O1C—B2C—C31C	64 (3)
O1A—B1A—C1A—C2A	−174.7 (6)	O1C—B1C—C1C—C2C	−170.6 (6)
C11A—B1A—C1A—C2A	2.5 (10)	C11C—B1C—C1C—C2C	8.2 (10)
O1A—B1A—C1A—C6A	5.1 (10)	O1C—B1C—C1C—C6C	9.3 (10)
C11A—B1A—C1A—C6A	−177.8 (6)	C11C—B1C—C1C—C6C	−171.8 (6)
C6A—C1A—C2A—C3A	−1.8 (10)	C6C—C1C—C2C—C3C	−1.5 (9)
B1A—C1A—C2A—C3A	178.0 (6)	B1C—C1C—C2C—C3C	178.5 (7)
C6A—C1A—C2A—S1A	177.7 (5)	C6C—C1C—C2C—S1C	−179.5 (6)
B1A—C1A—C2A—S1A	−2.6 (9)	B1C—C1C—C2C—S1C	0.5 (9)
C12A—S1A—C2A—C1A	0.7 (7)	C12C—S1C—C2C—C1C	−6.9 (7)
C12A—S1A—C2A—C3A	−179.9 (5)	C12C—S1C—C2C—C3C	175.0 (5)
C1A—C2A—C3A—C4A	1.7 (10)	C1C—C2C—C3C—C4C	0.9 (11)
S1A—C2A—C3A—C4A	−177.8 (6)	S1C—C2C—C3C—C4C	179.1 (6)
C2A—C3A—C4A—C5A	−1.1 (11)	C2C—C3C—C4C—C5C	0.2 (12)
C3A—C4A—C5A—C6A	0.6 (12)	C3C—C4C—C5C—C6C	−0.6 (13)
C4A—C5A—C6A—C1A	−0.8 (12)	C4C—C5C—C6C—C1C	0.0 (12)
C2A—C1A—C6A—C5A	1.3 (11)	C2C—C1C—C6C—C5C	1.1 (11)
B1A—C1A—C6A—C5A	−178.4 (7)	B1C—C1C—C6C—C5C	−178.9 (8)
O1A—B1A—C11A—C16A	−4.7 (10)	O1C—B1C—C11C—C12C	169.8 (6)
C1A—B1A—C11A—C16A	178.1 (7)	C1C—B1C—C11C—C12C	−9.0 (10)
O1A—B1A—C11A—C12A	177.0 (6)	O1C—B1C—C11C—C16C	−7.3 (10)
C1A—B1A—C11A—C12A	−0.2 (9)	C1C—B1C—C11C—C16C	173.9 (7)
C16A—C11A—C12A—C13A	−0.8 (9)	C16C—C11C—C12C—C13C	−0.1 (10)
B1A—C11A—C12A—C13A	177.6 (6)	B1C—C11C—C12C—C13C	−177.4 (7)
C16A—C11A—C12A—S1A	179.8 (5)	C16C—C11C—C12C—S1C	178.3 (6)
B1A—C11A—C12A—S1A	−1.8 (9)	B1C—C11C—C12C—S1C	1.1 (9)
C2A—S1A—C12A—C13A	−177.8 (5)	C2C—S1C—C12C—C11C	6.0 (7)
C2A—S1A—C12A—C11A	1.6 (6)	C2C—S1C—C12C—C13C	−175.5 (5)
C11A—C12A—C13A—C14A	0.1 (10)	C11C—C12C—C13C—C14C	−0.2 (11)
S1A—C12A—C13A—C14A	179.5 (5)	S1C—C12C—C13C—C14C	−178.8 (6)
C12A—C13A—C14A—C15A	0.6 (11)	C12C—C13C—C14C—C15C	−0.9 (12)
C13A—C14A—C15A—C16A	−0.6 (11)	C13C—C14C—C15C—C16C	2.3 (13)
C14A—C15A—C16A—C11A	−0.2 (12)	C14C—C15C—C16C—C11C	−2.7 (13)
C12A—C11A—C16A—C15A	0.9 (11)	C12C—C11C—C16C—C15C	1.6 (11)
B1A—C11A—C16A—C15A	−177.5 (7)	B1C—C11C—C16C—C15C	178.8 (7)
O1A—B2A—C21A—C26A	−0.9 (10)	O1C—B2C—C21C—C26C	−4.1 (11)
C31A—B2A—C21A—C26A	−179.6 (7)	C31C—B2C—C21C—C26C	176.1 (7)
O1A—B2A—C21A—C22A	179.5 (6)	O1C—B2C—C21C—C22C	179.2 (7)
C31A—B2A—C21A—C22A	0.8 (10)	C31C—B2C—C21C—C22C	−0.6 (11)
C26A—C21A—C22A—C23A	1.8 (10)	C26C—C21C—C22C—C23C	2.7 (10)
B2A—C21A—C22A—C23A	−178.5 (7)	B2C—C21C—C22C—C23C	179.6 (7)
C26A—C21A—C22A—S2A	−176.6 (5)	C26C—C21C—C22C—S2C	−177.8 (6)
B2A—C21A—C22A—S2A	3.0 (9)	B2C—C21C—C22C—S2C	−0.9 (10)
C32A—S2A—C22A—C23A	177.8 (5)	C32C—S2C—C22C—C23C	−179.2 (6)
C32A—S2A—C22A—C21A	−3.6 (7)	C32C—S2C—C22C—C21C	1.4 (7)
C21A—C22A—C23A—C24A	−2.9 (11)	C21C—C22C—C23C—C24C	−1.7 (11)
S2A—C22A—C23A—C24A	175.7 (6)	S2C—C22C—C23C—C24C	178.8 (6)
C22A—C23A—C24A—C25A	2.3 (12)	C22C—C23C—C24C—C25C	0.6 (12)
C23A—C24A—C25A—C26A	−0.8 (12)	C23C—C24C—C25C—C26C	−0.7 (12)
C24A—C25A—C26A—C21A	−0.2 (12)	C24C—C25C—C26C—C21C	1.9 (12)
C22A—C21A—C26A—C25A	−0.3 (10)	C22C—C21C—C26C—C25C	−2.8 (11)
B2A—C21A—C26A—C25A	−179.9 (7)	B2C—C21C—C26C—C25C	−179.7 (8)
O1A—B2A—C31A—C32A	177.5 (6)	O1C—B2C—C31C—C32C	−178.3 (7)
C21A—B2A—C31A—C32A	−3.8 (10)	C21C—B2C—C31C—C32C	1.4 (11)
O1A—B2A—C31A—C36A	−0.7 (10)	O1C—B2C—C31C—C36C	1.8 (11)
C21A—B2A—C31A—C36A	178.0 (6)	C21C—B2C—C31C—C36C	−178.5 (7)
C36A—C31A—C32A—C33A	0.6 (10)	C36C—C31C—C32C—C33C	−1.3 (10)
B2A—C31A—C32A—C33A	−177.7 (7)	B2C—C31C—C32C—C33C	178.8 (7)
C36A—C31A—C32A—S2A	−179.0 (5)	C36C—C31C—C32C—S2C	179.2 (5)
B2A—C31A—C32A—S2A	2.8 (10)	B2C—C31C—C32C—S2C	−0.7 (10)
C22A—S2A—C32A—C31A	0.7 (7)	C22C—S2C—C32C—C31C	−0.5 (7)
C22A—S2A—C32A—C33A	−178.8 (5)	C22C—S2C—C32C—C33C	179.9 (6)
C31A—C32A—C33A—C34A	−0.4 (11)	C31C—C32C—C33C—C34C	1.2 (11)
S2A—C32A—C33A—C34A	179.2 (6)	S2C—C32C—C33C—C34C	−179.2 (6)
C32A—C33A—C34A—C35A	−0.4 (12)	C32C—C33C—C34C—C35C	−0.6 (11)
C33A—C34A—C35A—C36A	1.0 (11)	C33C—C34C—C35C—C36C	0.1 (11)
C34A—C35A—C36A—C31A	−0.8 (10)	C34C—C35C—C36C—C31C	−0.3 (11)
C32A—C31A—C36A—C35A	0.0 (10)	C32C—C31C—C36C—C35C	0.8 (11)
B2A—C31A—C36A—C35A	178.2 (6)	B2C—C31C—C36C—C35C	−179.2 (7)
B2B—O1B—B1B—C1B	−122 (2)	B2D—O1D—B1D—C1D	−41 (3)
B2B—O1B—B1B—C11B	60 (3)	B2D—O1D—B1D—C11D	138 (3)
B1B—O1B—B2B—C31B	−149 (2)	B1D—O1D—B2D—C31D	132 (3)
B1B—O1B—B2B—C21B	30 (3)	B1D—O1D—B2D—C21D	−50 (3)
O1B—B1B—C1B—C2B	−179.8 (7)	O1D—B1D—C1D—C2D	−177.2 (7)
C11B—B1B—C1B—C2B	−2.1 (11)	C11D—B1D—C1D—C2D	3.5 (11)
O1B—B1B—C1B—C6B	0.2 (11)	O1D—B1D—C1D—C6D	1.3 (11)
C11B—B1B—C1B—C6B	177.9 (6)	C11D—B1D—C1D—C6D	−178.0 (7)
C6B—C1B—C2B—C3B	−0.5 (10)	C6D—C1D—C2D—C3D	−1.9 (10)
B1B—C1B—C2B—C3B	179.6 (7)	B1D—C1D—C2D—C3D	176.8 (7)
C6B—C1B—C2B—S1B	179.7 (5)	C6D—C1D—C2D—S1D	178.7 (6)
B1B—C1B—C2B—S1B	−0.3 (10)	B1D—C1D—C2D—S1D	−2.7 (10)
C12B—S1B—C2B—C3B	−178.3 (5)	C12D—S1D—C2D—C1D	0.4 (7)
C12B—S1B—C2B—C1B	1.6 (7)	C12D—S1D—C2D—C3D	−179.1 (6)
C1B—C2B—C3B—C4B	−0.3 (11)	C1D—C2D—C3D—C4D	1.1 (12)
S1B—C2B—C3B—C4B	179.5 (6)	S1D—C2D—C3D—C4D	−179.4 (6)
C2B—C3B—C4B—C5B	0.6 (11)	C2D—C3D—C4D—C5D	0.3 (12)
C3B—C4B—C5B—C6B	−0.1 (12)	C3D—C4D—C5D—C6D	−0.7 (12)
C4B—C5B—C6B—C1B	−0.8 (11)	C4D—C5D—C6D—C1D	−0.2 (11)
C2B—C1B—C6B—C5B	1.0 (10)	C2D—C1D—C6D—C5D	1.4 (10)
B1B—C1B—C6B—C5B	−179.0 (7)	B1D—C1D—C6D—C5D	−177.2 (7)
O1B—B1B—C11B—C12B	−179.3 (7)	O1D—B1D—C11D—C12D	178.9 (7)
C1B—B1B—C11B—C12B	3.0 (11)	C1D—B1D—C11D—C12D	−1.8 (11)
O1B—B1B—C11B—C16B	−0.1 (11)	O1D—B1D—C11D—C16D	0.1 (11)
C1B—B1B—C11B—C16B	−177.9 (7)	C1D—B1D—C11D—C16D	179.5 (7)
C16B—C11B—C12B—C13B	1.4 (10)	C16D—C11D—C12D—C13D	−2.0 (10)
B1B—C11B—C12B—C13B	−179.5 (7)	B1D—C11D—C12D—C13D	179.2 (7)
C16B—C11B—C12B—S1B	179.4 (5)	C16D—C11D—C12D—S1D	178.2 (6)
B1B—C11B—C12B—S1B	−1.5 (10)	B1D—C11D—C12D—S1D	−0.6 (10)
C2B—S1B—C12B—C11B	−0.7 (7)	C2D—S1D—C12D—C11D	1.2 (7)
C2B—S1B—C12B—C13B	177.5 (5)	C2D—S1D—C12D—C13D	−178.6 (5)
C11B—C12B—C13B—C14B	−1.7 (11)	C11D—C12D—C13D—C14D	2.0 (11)
S1B—C12B—C13B—C14B	−179.9 (6)	S1D—C12D—C13D—C14D	−178.1 (6)
C12B—C13B—C14B—C15B	1.7 (12)	C12D—C13D—C14D—C15D	−1.0 (12)
C13B—C14B—C15B—C16B	−1.5 (12)	C13D—C14D—C15D—C16D	0.1 (14)
C14B—C15B—C16B—C11B	1.2 (12)	C14D—C15D—C16D—C11D	−0.1 (13)
C12B—C11B—C16B—C15B	−1.1 (11)	C12D—C11D—C16D—C15D	1.1 (11)
B1B—C11B—C16B—C15B	179.7 (7)	B1D—C11D—C16D—C15D	179.8 (7)
O1B—B2B—C21B—C22B	−178.2 (7)	O1D—B2D—C21D—C22D	179.9 (6)
C31B—B2B—C21B—C22B	1.4 (10)	C31D—B2D—C21D—C22D	−2.0 (10)
O1B—B2B—C21B—C26B	2.6 (10)	O1D—B2D—C21D—C26D	0.8 (11)
C31B—B2B—C21B—C26B	−177.9 (7)	C31D—B2D—C21D—C26D	178.9 (7)
C26B—C21B—C22B—C23B	−0.4 (10)	C26D—C21D—C22D—C23D	−1.1 (10)
B2B—C21B—C22B—C23B	−179.7 (7)	B2D—C21D—C22D—C23D	179.7 (7)
C26B—C21B—C22B—S2B	−178.2 (6)	C26D—C21D—C22D—S2D	177.8 (6)
B2B—C21B—C22B—S2B	2.5 (9)	B2D—C21D—C22D—S2D	−1.4 (9)
C32B—S2B—C22B—C21B	−4.3 (7)	C32D—S2D—C22D—C21D	3.1 (6)
C32B—S2B—C22B—C23B	177.8 (5)	C32D—S2D—C22D—C23D	−177.9 (5)
C21B—C22B—C23B—C24B	−0.1 (11)	C21D—C22D—C23D—C24D	−0.5 (10)
S2B—C22B—C23B—C24B	177.9 (6)	S2D—C22D—C23D—C24D	−179.6 (6)
C22B—C23B—C24B—C25B	0.3 (12)	C22D—C23D—C24D—C25D	2.2 (11)
C23B—C24B—C25B—C26B	0.0 (13)	C23D—C24D—C25D—C26D	−2.1 (12)
C24B—C25B—C26B—C21B	−0.6 (13)	C24D—C25D—C26D—C21D	0.3 (12)
C22B—C21B—C26B—C25B	0.8 (11)	C22D—C21D—C26D—C25D	1.2 (11)
B2B—C21B—C26B—C25B	−180.0 (7)	B2D—C21D—C26D—C25D	−179.6 (7)
O1B—B2B—C31B—C32B	176.8 (6)	O1D—B2D—C31D—C36D	−0.9 (10)
C21B—B2B—C31B—C32B	−2.8 (10)	C21D—B2D—C31D—C36D	−179.0 (7)
O1B—B2B—C31B—C36B	−3.3 (10)	O1D—B2D—C31D—C32D	−178.9 (6)
C21B—B2B—C31B—C36B	177.2 (7)	C21D—B2D—C31D—C32D	3.0 (10)
C36B—C31B—C32B—C33B	1.1 (10)	C36D—C31D—C32D—C33D	1.6 (10)
B2B—C31B—C32B—C33B	−178.9 (7)	B2D—C31D—C32D—C33D	179.8 (7)
C36B—C31B—C32B—S2B	−179.6 (5)	C36D—C31D—C32D—S2D	−178.7 (6)
B2B—C31B—C32B—S2B	0.3 (9)	B2D—C31D—C32D—S2D	−0.6 (9)
C22B—S2B—C32B—C33B	−177.9 (5)	C22D—S2D—C32D—C31D	−2.1 (7)
C22B—S2B—C32B—C31B	2.8 (6)	C22D—S2D—C32D—C33D	177.6 (5)
C31B—C32B—C33B—C34B	−0.4 (11)	C31D—C32D—C33D—C34D	−1.1 (11)
S2B—C32B—C33B—C34B	−179.7 (6)	S2D—C32D—C33D—C34D	179.2 (6)
C32B—C33B—C34B—C35B	−0.5 (11)	C32D—C33D—C34D—C35D	−0.1 (12)
C33B—C34B—C35B—C36B	0.7 (12)	C33D—C34D—C35D—C36D	0.6 (12)
C34B—C35B—C36B—C31B	0.0 (12)	C34D—C35D—C36D—C31D	0.0 (12)
C32B—C31B—C36B—C35B	−0.9 (11)	C32D—C31D—C36D—C35D	−1.1 (11)
B2B—C31B—C36B—C35B	179.1 (7)	B2D—C31D—C36D—C35D	−179.2 (7)

### 10,10'-Oxybis(9-thia-10-hydro-10-boraanthracene) (II) Crystal data

**Table:**

C_24_H_16_B_2_OS_2_	*F*(000) = 1680
*M**_r_* = 406.11	*D*_x_ = 1.381 Mg m^−^^3^
Monoclinic, *P*2_1_/*c*	Mo *K*α radiation, λ = 0.71073 Å
*a* = 23.2471 (18) Å	Cell parameters from 6545 reflections
*b* = 12.5949 (16) Å	θ = 2.3–23.9°
*c* = 13.3561 (11) Å	µ = 0.29 mm^−^^1^
β = 92.217 (6)°	*T* = 173 K
*V* = 3907.7 (7) Å^3^	Needle, colourless
*Z* = 8	0.19 × 0.12 × 0.09 mm

### 10,10'-Oxybis(9-thia-10-hydro-10-boraanthracene) (II) Data collection

**Table:**

Stoe IPDS II two-circle diffractometer	32689 independent reflections
Radiation source: Genix 3D IµS microfocus X-ray source	13195 reflections with *I* > 2σ(*I*)
ω scans	θ_max_ = 25.0°, θ_min_ = 2.4°
Absorption correction: multi-scan (X-Area; Stoe & Cie, 2001)	*h* = −27→27
*T*_min_ = 0.677, *T*_max_ = 1.000	*k* = −14→14
32689 measured reflections	*l* = −15→15

### 10,10'-Oxybis(9-thia-10-hydro-10-boraanthracene) (II) Refinement

**Table:**

Refinement on *F*^2^	Primary atom site location: structure-invariant direct methods
Least-squares matrix: full	Hydrogen site location: inferred from neighbouring sites
*R*[*F*^2^ > 2σ(*F*^2^)] = 0.079	H-atom parameters constrained
*wR*(*F*^2^) = 0.184	*w* = 1/[σ^2^(*F*_o_^2^) + (0.0581*P*)^2^] where *P* = (*F*_o_^2^ + 2*F*_c_^2^)/3
*S* = 0.86	(Δ/σ)_max_ < 0.001
32689 reflections	Δρ_max_ = 0.48 e Å^−^^3^
524 parameters	Δρ_min_ = −0.28 e Å^−^^3^
0 restraints	

### 10,10'-Oxybis(9-thia-10-hydro-10-boraanthracene) (II) Special details

**Table:**

Geometry. All esds (except the esd in the dihedral angle between two l.s. planes) are estimated using the full covariance matrix. The cell esds are taken into account individually in the estimation of esds in distances, angles and torsion angles; correlations between esds in cell parameters are only used when they are defined by crystal symmetry. An approximate (isotropic) treatment of cell esds is used for estimating esds involving l.s. planes.
Refinement. Refined as a 2-component twin.

### 10,10'-Oxybis(9-thia-10-hydro-10-boraanthracene) (II) Fractional atomic coordinates and isotropic or equivalent isotropic displacement parameters (Å^2^)

**Table:**

	*x*	*y*	*z*	*U*_iso_*/*U*_eq_	
S1	0.43516 (7)	0.40888 (17)	0.35977 (14)	0.0626 (5)	
S2	0.82281 (8)	0.37484 (16)	0.36230 (15)	0.0662 (5)	
B1	0.5722 (3)	0.4025 (8)	0.3877 (7)	0.062 (2)	
B2	0.6871 (4)	0.3869 (9)	0.3866 (8)	0.074 (3)	
O1	0.6303 (2)	0.3935 (6)	0.3941 (5)	0.111 (2)	
C1	0.5359 (3)	0.3507 (6)	0.4647 (6)	0.0570 (19)	
C2	0.4763 (3)	0.3519 (5)	0.4574 (5)	0.0520 (18)	
C3	0.4441 (3)	0.3024 (6)	0.5317 (5)	0.0613 (19)	
H3	0.4033	0.3031	0.5258	0.074*	
C4	0.4704 (4)	0.2541 (6)	0.6109 (6)	0.070 (2)	
H4	0.4480	0.2207	0.6599	0.084*	
C5	0.5290 (4)	0.2531 (7)	0.6210 (6)	0.077 (2)	
H5	0.5472	0.2192	0.6773	0.092*	
C6	0.5616 (3)	0.3000 (7)	0.5514 (7)	0.072 (2)	
H6	0.6024	0.2993	0.5604	0.087*	
C11	0.5436 (3)	0.4650 (6)	0.2981 (6)	0.060 (2)	
C12	0.4842 (3)	0.4719 (6)	0.2839 (6)	0.0580 (19)	
C13	0.4584 (4)	0.5294 (6)	0.2030 (6)	0.074 (2)	
H13	0.4177	0.5338	0.1944	0.089*	
C14	0.4934 (5)	0.5785 (7)	0.1376 (7)	0.091 (3)	
H14	0.4770	0.6155	0.0815	0.109*	
C15	0.5524 (5)	0.5751 (7)	0.1520 (8)	0.089 (3)	
H15	0.5760	0.6134	0.1081	0.107*	
C16	0.5768 (4)	0.5181 (7)	0.2273 (7)	0.076 (2)	
H16	0.6176	0.5134	0.2330	0.092*	
C21	0.7261 (3)	0.4665 (6)	0.4404 (6)	0.065 (2)	
C22	0.7854 (3)	0.4663 (6)	0.4341 (6)	0.0612 (19)	
C23	0.8199 (4)	0.5424 (7)	0.4834 (6)	0.081 (2)	
H23	0.8604	0.5422	0.4767	0.098*	
C24	0.7940 (7)	0.6184 (8)	0.5424 (8)	0.114 (4)	
H24	0.8172	0.6702	0.5763	0.137*	
C25	0.7353 (7)	0.6198 (10)	0.5525 (7)	0.120 (5)	
H25	0.7179	0.6704	0.5945	0.144*	
C26	0.7032 (5)	0.5474 (9)	0.5011 (7)	0.093 (3)	
H26	0.6626	0.5508	0.5059	0.111*	
C31	0.7129 (3)	0.2958 (6)	0.3196 (5)	0.0561 (19)	
C32	0.7713 (3)	0.2881 (6)	0.3083 (5)	0.0490 (17)	
C33	0.7945 (3)	0.2069 (7)	0.2520 (6)	0.070 (2)	
H33	0.8349	0.2025	0.2454	0.084*	
C34	0.7590 (5)	0.1329 (7)	0.2057 (7)	0.082 (3)	
H34	0.7752	0.0786	0.1663	0.098*	
C35	0.7018 (5)	0.1363 (8)	0.2154 (8)	0.088 (3)	
H35	0.6777	0.0849	0.1829	0.106*	
C36	0.6780 (4)	0.2161 (8)	0.2737 (7)	0.080 (3)	
H36	0.6377	0.2169	0.2827	0.096*	
O1A	0.1319 (2)	0.4253 (5)	0.1099 (5)	0.0859 (18)	
B1A	0.1859 (4)	0.3986 (7)	0.1160 (6)	0.057 (2)	
B2A	0.0740 (4)	0.4187 (7)	0.1190 (7)	0.060 (2)	
S1A	0.32048 (9)	0.3448 (2)	0.1355 (2)	0.0927 (8)	
S2A	−0.06322 (7)	0.40963 (15)	0.13954 (14)	0.0575 (5)	
C1A	0.2075 (3)	0.2985 (6)	0.1766 (6)	0.0607 (19)	
C2A	0.2660 (3)	0.2748 (6)	0.1860 (6)	0.070 (2)	
C3A	0.2841 (6)	0.1879 (9)	0.2445 (8)	0.110 (4)	
H3A	0.3241	0.1737	0.2536	0.132*	
C4A	0.2448 (9)	0.1223 (11)	0.2894 (9)	0.146 (7)	
H4A	0.2572	0.0615	0.3264	0.175*	
C5A	0.1879 (7)	0.1474 (9)	0.2791 (7)	0.118 (4)	
H5A	0.1608	0.1038	0.3111	0.142*	
C6A	0.1685 (4)	0.2315 (8)	0.2253 (7)	0.085 (3)	
H6A	0.1284	0.2459	0.2199	0.102*	
C11A	0.2300 (3)	0.4673 (6)	0.0636 (5)	0.0581 (19)	
C12A	0.2880 (3)	0.4474 (7)	0.0666 (5)	0.064 (2)	
C13A	0.3269 (5)	0.5114 (12)	0.0160 (10)	0.117 (4)	
H13A	0.3667	0.4939	0.0186	0.140*	
C14A	0.3094 (10)	0.5935 (16)	−0.0338 (11)	0.165 (9)	
H14A	0.3364	0.6362	−0.0671	0.198*	
C15A	0.2528 (9)	0.6196 (11)	−0.0393 (9)	0.139 (6)	
H15A	0.2407	0.6814	−0.0750	0.167*	
C16A	0.2125 (5)	0.5574 (8)	0.0061 (7)	0.096 (3)	
H16A	0.1729	0.5750	−0.0011	0.115*	
C21A	0.0463 (3)	0.4731 (5)	0.2099 (5)	0.0517 (17)	
C22A	−0.0127 (3)	0.4733 (5)	0.2212 (5)	0.0510 (17)	
C23A	−0.0368 (3)	0.5254 (6)	0.3030 (6)	0.063 (2)	
H23A	−0.0773	0.5254	0.3098	0.076*	
C24A	−0.0018 (4)	0.5758 (6)	0.3724 (6)	0.072 (2)	
H24A	−0.0184	0.6117	0.4269	0.086*	
C25A	0.0569 (4)	0.5755 (6)	0.3645 (6)	0.073 (2)	
H25A	0.0813	0.6094	0.4135	0.087*	
C26A	0.0794 (3)	0.5247 (6)	0.2839 (7)	0.072 (2)	
H26A	0.1200	0.5249	0.2784	0.086*	
C31A	0.0356 (3)	0.3593 (5)	0.0389 (5)	0.0499 (17)	
C32A	−0.0237 (3)	0.3539 (5)	0.0422 (5)	0.0466 (16)	
C33A	−0.0569 (3)	0.3003 (6)	−0.0300 (6)	0.0596 (19)	
H33A	−0.0976	0.2969	−0.0255	0.072*	
C34A	−0.0305 (4)	0.2526 (6)	−0.1077 (6)	0.065 (2)	
H34A	−0.0530	0.2169	−0.1581	0.078*	
C35A	0.0280 (4)	0.2555 (7)	−0.1138 (6)	0.072 (2)	
H35A	0.0461	0.2205	−0.1671	0.087*	
C36A	0.0601 (3)	0.3090 (6)	−0.0425 (7)	0.067 (2)	
H36A	0.1007	0.3122	−0.0485	0.080*	

### 10,10'-Oxybis(9-thia-10-hydro-10-boraanthracene) (II) Atomic displacement parameters (Å^2^)

**Table:**

	*U*^11^	*U*^22^	*U*^33^	*U*^12^	*U*^13^	*U*^23^
S1	0.0533 (11)	0.0670 (13)	0.0674 (13)	0.0100 (9)	0.0028 (8)	0.0037 (11)
S2	0.0521 (11)	0.0658 (13)	0.0808 (14)	−0.0020 (9)	0.0062 (8)	−0.0091 (11)
B1	0.052 (5)	0.063 (6)	0.072 (6)	0.008 (4)	0.005 (4)	−0.008 (5)
B2	0.054 (6)	0.089 (8)	0.080 (7)	0.017 (5)	0.014 (4)	0.041 (6)
O1	0.049 (3)	0.160 (7)	0.126 (6)	0.033 (4)	0.019 (3)	0.036 (5)
C1	0.053 (4)	0.053 (5)	0.065 (5)	0.006 (3)	0.001 (3)	−0.006 (4)
C2	0.054 (4)	0.041 (4)	0.061 (5)	0.008 (3)	0.002 (3)	0.000 (3)
C3	0.070 (5)	0.055 (5)	0.058 (5)	0.009 (4)	0.002 (3)	0.015 (4)
C4	0.075 (6)	0.056 (5)	0.080 (6)	−0.009 (4)	0.008 (4)	0.003 (4)
C5	0.098 (7)	0.065 (5)	0.066 (5)	−0.006 (5)	−0.011 (4)	0.022 (5)
C6	0.060 (5)	0.062 (6)	0.094 (7)	0.011 (4)	−0.013 (4)	0.000 (5)
C11	0.058 (5)	0.055 (5)	0.069 (5)	−0.008 (4)	0.022 (4)	−0.015 (4)
C12	0.063 (5)	0.041 (5)	0.071 (5)	0.003 (3)	0.006 (3)	−0.009 (4)
C13	0.090 (6)	0.056 (5)	0.076 (6)	0.008 (4)	0.000 (4)	0.003 (5)
C14	0.129 (9)	0.061 (6)	0.083 (7)	0.007 (6)	0.009 (5)	0.011 (5)
C15	0.121 (8)	0.060 (6)	0.088 (7)	−0.007 (6)	0.032 (6)	0.000 (5)
C16	0.073 (5)	0.064 (6)	0.094 (7)	−0.007 (4)	0.019 (4)	0.001 (5)
C21	0.076 (6)	0.060 (5)	0.059 (5)	0.017 (4)	0.007 (4)	0.014 (4)
C22	0.077 (5)	0.048 (5)	0.058 (5)	0.000 (4)	0.007 (4)	0.010 (4)
C23	0.111 (7)	0.074 (6)	0.058 (5)	−0.022 (5)	0.001 (4)	0.003 (5)
C24	0.228 (13)	0.058 (7)	0.057 (6)	−0.021 (8)	0.006 (7)	−0.011 (5)
C25	0.241 (15)	0.079 (9)	0.041 (6)	0.053 (9)	0.028 (8)	0.010 (5)
C26	0.132 (8)	0.090 (8)	0.058 (6)	0.052 (6)	0.022 (5)	0.009 (5)
C31	0.046 (4)	0.076 (6)	0.045 (4)	−0.007 (4)	−0.009 (3)	0.020 (4)
C32	0.054 (4)	0.047 (4)	0.046 (4)	0.000 (3)	−0.005 (3)	0.010 (3)
C33	0.070 (5)	0.057 (6)	0.082 (6)	0.009 (4)	0.002 (4)	−0.004 (4)
C34	0.123 (8)	0.056 (6)	0.066 (6)	0.003 (6)	−0.010 (5)	−0.009 (4)
C35	0.116 (8)	0.062 (7)	0.085 (7)	−0.032 (6)	−0.022 (5)	0.002 (5)
C36	0.066 (5)	0.093 (7)	0.081 (6)	−0.024 (5)	−0.013 (4)	0.037 (6)
O1A	0.047 (3)	0.097 (5)	0.113 (5)	0.014 (3)	0.000 (3)	−0.017 (4)
B1A	0.054 (5)	0.060 (6)	0.057 (5)	−0.002 (4)	0.002 (4)	−0.020 (4)
B2A	0.064 (6)	0.043 (5)	0.072 (6)	0.010 (4)	−0.005 (4)	0.010 (4)
S1A	0.0523 (13)	0.109 (2)	0.116 (2)	0.0165 (12)	0.0001 (11)	−0.0204 (16)
S2A	0.0587 (11)	0.0531 (12)	0.0611 (12)	0.0003 (9)	0.0078 (8)	−0.0040 (10)
C1A	0.067 (5)	0.054 (5)	0.062 (5)	−0.007 (4)	0.004 (3)	−0.010 (4)
C2A	0.078 (6)	0.055 (5)	0.075 (6)	0.015 (4)	0.000 (4)	−0.020 (4)
C3A	0.177 (11)	0.057 (7)	0.092 (8)	0.044 (7)	−0.031 (7)	0.009 (6)
C4A	0.31 (2)	0.051 (8)	0.070 (8)	0.040 (11)	−0.040 (11)	−0.016 (6)
C5A	0.256 (16)	0.060 (8)	0.039 (5)	−0.036 (9)	0.017 (7)	−0.006 (5)
C6A	0.124 (7)	0.071 (7)	0.063 (5)	−0.031 (5)	0.029 (5)	−0.021 (5)
C11A	0.083 (6)	0.055 (5)	0.036 (4)	0.002 (4)	−0.002 (3)	0.002 (3)
C12A	0.065 (5)	0.079 (6)	0.048 (4)	−0.015 (4)	0.010 (3)	−0.009 (4)
C13A	0.119 (9)	0.130 (11)	0.103 (9)	−0.056 (8)	0.037 (7)	−0.041 (8)
C14A	0.29 (2)	0.134 (16)	0.079 (10)	−0.109 (18)	0.047 (13)	−0.040 (10)
C15A	0.294 (19)	0.057 (7)	0.065 (8)	−0.037 (12)	−0.014 (11)	0.014 (6)
C16A	0.135 (8)	0.071 (7)	0.080 (7)	0.002 (6)	−0.010 (6)	−0.012 (6)
C21A	0.056 (5)	0.042 (4)	0.057 (4)	−0.004 (3)	−0.009 (3)	0.003 (3)
C22A	0.066 (5)	0.037 (4)	0.050 (4)	−0.005 (3)	0.001 (3)	−0.001 (3)
C23A	0.078 (5)	0.052 (5)	0.061 (5)	−0.007 (4)	0.015 (4)	0.002 (4)
C24A	0.119 (7)	0.052 (5)	0.045 (5)	−0.011 (5)	0.010 (4)	−0.019 (4)
C25A	0.107 (7)	0.052 (5)	0.058 (5)	−0.014 (5)	−0.008 (4)	−0.020 (4)
C26A	0.062 (5)	0.056 (6)	0.095 (6)	−0.012 (4)	−0.013 (4)	0.007 (5)
C31A	0.064 (4)	0.035 (4)	0.051 (4)	0.006 (3)	0.015 (3)	0.002 (3)
C32A	0.050 (4)	0.039 (4)	0.051 (4)	0.008 (3)	0.007 (3)	0.002 (3)
C33A	0.059 (4)	0.051 (5)	0.070 (5)	0.002 (3)	0.003 (3)	0.004 (4)
C34A	0.086 (6)	0.057 (5)	0.054 (5)	−0.005 (4)	0.009 (4)	−0.002 (4)
C35A	0.093 (7)	0.058 (5)	0.067 (6)	−0.002 (5)	0.026 (4)	−0.006 (4)
C36A	0.061 (5)	0.047 (5)	0.095 (7)	0.004 (4)	0.023 (4)	0.004 (4)

### 10,10'-Oxybis(9-thia-10-hydro-10-boraanthracene) (II) Geometric parameters (Å, º)

**Table:**

S1—C12	1.744 (8)	O1A—B1A	1.300 (9)
S1—C2	1.742 (7)	O1A—B2A	1.357 (9)
S2—C22	1.751 (8)	B1A—C11A	1.531 (11)
S2—C32	1.755 (7)	B1A—C1A	1.571 (12)
B1—O1	1.356 (9)	B2A—C21A	1.556 (11)
B1—C1	1.504 (12)	B2A—C31A	1.559 (12)
B1—C11	1.559 (12)	S1A—C2A	1.704 (9)
B2—O1	1.330 (9)	S1A—C12A	1.743 (9)
B2—C21	1.515 (14)	S2A—C22A	1.764 (7)
B2—C31	1.586 (13)	S2A—C32A	1.766 (6)
C1—C2	1.385 (8)	C1A—C2A	1.392 (9)
C1—C6	1.432 (11)	C1A—C6A	1.416 (11)
C2—C3	1.411 (10)	C2A—C3A	1.400 (13)
C3—C4	1.345 (10)	C3A—C4A	1.385 (18)
C3—H3	0.9500	C3A—H3A	0.9500
C4—C5	1.365 (10)	C4A—C5A	1.363 (16)
C4—H4	0.9500	C4A—H4A	0.9500
C5—C6	1.357 (11)	C5A—C6A	1.348 (15)
C5—H5	0.9500	C5A—H5A	0.9500
C6—H6	0.9500	C6A—H6A	0.9500
C11—C12	1.391 (9)	C11A—C12A	1.370 (9)
C11—C16	1.413 (11)	C11A—C16A	1.421 (12)
C12—C13	1.416 (11)	C12A—C13A	1.405 (13)
C13—C14	1.366 (12)	C13A—C14A	1.29 (2)
C13—H13	0.9500	C13A—H13A	0.9500
C14—C15	1.377 (12)	C14A—C15A	1.357 (19)
C14—H14	0.9500	C14A—H14A	0.9500
C15—C16	1.343 (12)	C15A—C16A	1.378 (17)
C15—H15	0.9500	C15A—H15A	0.9500
C16—H16	0.9500	C16A—H16A	0.9500
C21—C22	1.384 (9)	C21A—C22A	1.385 (8)
C21—C26	1.419 (12)	C21A—C26A	1.390 (10)
C22—C23	1.398 (11)	C22A—C23A	1.409 (10)
C23—C24	1.392 (14)	C23A—C24A	1.366 (10)
C23—H23	0.9500	C23A—H23A	0.9500
C24—C25	1.375 (14)	C24A—C25A	1.373 (10)
C24—H24	0.9500	C24A—H24A	0.9500
C25—C26	1.349 (15)	C25A—C26A	1.373 (11)
C25—H25	0.9500	C25A—H25A	0.9500
C26—H26	0.9500	C26A—H26A	0.9500
C31—C32	1.375 (8)	C31A—C32A	1.382 (8)
C31—C36	1.415 (12)	C31A—C36A	1.399 (10)
C32—C33	1.391 (10)	C32A—C33A	1.387 (10)
C33—C34	1.375 (11)	C33A—C34A	1.366 (10)
C33—H33	0.9500	C33A—H33A	0.9500
C34—C35	1.340 (11)	C34A—C35A	1.365 (10)
C34—H34	0.9500	C34A—H34A	0.9500
C35—C36	1.400 (13)	C35A—C36A	1.365 (11)
C35—H35	0.9500	C35A—H35A	0.9500
C36—H36	0.9500	C36A—H36A	0.9500
			
C12—S1—C2	105.7 (3)	B1A—O1A—B2A	159.5 (7)
C22—S2—C32	106.8 (4)	O1A—B1A—C11A	119.2 (8)
O1—B1—C1	120.3 (8)	O1A—B1A—C1A	122.0 (7)
O1—B1—C11	118.9 (7)	C11A—B1A—C1A	118.8 (7)
C1—B1—C11	120.7 (6)	O1A—B2A—C21A	119.0 (7)
O1—B2—C21	120.0 (9)	O1A—B2A—C31A	120.8 (7)
O1—B2—C31	119.1 (9)	C21A—B2A—C31A	120.2 (7)
C21—B2—C31	120.9 (7)	C2A—S1A—C12A	106.2 (4)
B2—O1—B1	172.0 (7)	C22A—S2A—C32A	106.4 (3)
C2—C1—C6	116.6 (6)	C2A—C1A—C6A	118.1 (8)
C2—C1—B1	122.2 (7)	C2A—C1A—B1A	120.7 (6)
C6—C1—B1	121.2 (7)	C6A—C1A—B1A	121.1 (8)
C1—C2—C3	120.1 (6)	C1A—C2A—C3A	119.4 (9)
C1—C2—S1	125.2 (5)	C1A—C2A—S1A	126.2 (6)
C3—C2—S1	114.7 (5)	C3A—C2A—S1A	114.4 (8)
C4—C3—C2	121.0 (7)	C4A—C3A—C2A	121.2 (12)
C4—C3—H3	119.5	C4A—C3A—H3A	119.4
C2—C3—H3	119.5	C2A—C3A—H3A	119.4
C3—C4—C5	120.3 (7)	C5A—C4A—C3A	118.2 (12)
C3—C4—H4	119.9	C5A—C4A—H4A	120.9
C5—C4—H4	119.9	C3A—C4A—H4A	120.9
C6—C5—C4	120.7 (8)	C6A—C5A—C4A	122.7 (12)
C6—C5—H5	119.7	C6A—C5A—H5A	118.7
C4—C5—H5	119.7	C4A—C5A—H5A	118.7
C5—C6—C1	121.4 (7)	C5A—C6A—C1A	120.4 (10)
C5—C6—H6	119.3	C5A—C6A—H6A	119.8
C1—C6—H6	119.3	C1A—C6A—H6A	119.8
C12—C11—C16	116.5 (8)	C12A—C11A—C16A	114.9 (7)
C12—C11—B1	121.8 (6)	C12A—C11A—B1A	124.0 (7)
C16—C11—B1	121.7 (7)	C16A—C11A—B1A	121.1 (8)
C11—C12—C13	121.7 (7)	C11A—C12A—C13A	122.2 (10)
C11—C12—S1	124.1 (6)	C11A—C12A—S1A	123.9 (6)
C13—C12—S1	114.2 (6)	C13A—C12A—S1A	113.9 (9)
C14—C13—C12	118.3 (8)	C14A—C13A—C12A	120.8 (15)
C14—C13—H13	120.9	C14A—C13A—H13A	119.6
C12—C13—H13	120.9	C12A—C13A—H13A	119.6
C13—C14—C15	120.8 (9)	C13A—C14A—C15A	120.6 (17)
C13—C14—H14	119.6	C13A—C14A—H14A	119.7
C15—C14—H14	119.6	C15A—C14A—H14A	119.7
C16—C15—C14	120.7 (8)	C14A—C15A—C16A	120.8 (15)
C16—C15—H15	119.7	C14A—C15A—H15A	119.6
C14—C15—H15	119.7	C16A—C15A—H15A	119.6
C15—C16—C11	121.9 (8)	C15A—C16A—C11A	120.5 (11)
C15—C16—H16	119.1	C15A—C16A—H16A	119.8
C11—C16—H16	119.1	C11A—C16A—H16A	119.8
C22—C21—C26	115.6 (8)	C22A—C21A—C26A	116.4 (7)
C22—C21—B2	123.3 (7)	C22A—C21A—B2A	121.8 (6)
C26—C21—B2	121.1 (8)	C26A—C21A—B2A	121.8 (7)
C21—C22—C23	121.6 (8)	C21A—C22A—C23A	120.6 (6)
C21—C22—S2	123.5 (6)	C21A—C22A—S2A	124.8 (5)
C23—C22—S2	114.9 (6)	C23A—C22A—S2A	114.6 (5)
C24—C23—C22	119.1 (9)	C24A—C23A—C22A	120.0 (7)
C24—C23—H23	120.5	C24A—C23A—H23A	120.0
C22—C23—H23	120.5	C22A—C23A—H23A	120.0
C25—C24—C23	121.1 (10)	C23A—C24A—C25A	120.9 (7)
C25—C24—H24	119.4	C23A—C24A—H24A	119.6
C23—C24—H24	119.4	C25A—C24A—H24A	119.6
C26—C25—C24	118.2 (10)	C26A—C25A—C24A	118.1 (7)
C26—C25—H25	120.9	C26A—C25A—H25A	120.9
C24—C25—H25	120.9	C24A—C25A—H25A	120.9
C25—C26—C21	124.4 (10)	C25A—C26A—C21A	124.0 (8)
C25—C26—H26	117.8	C25A—C26A—H26A	118.0
C21—C26—H26	117.8	C21A—C26A—H26A	118.0
C32—C31—C36	117.0 (8)	C32A—C31A—C36A	116.1 (6)
C32—C31—B2	120.6 (7)	C32A—C31A—B2A	123.2 (6)
C36—C31—B2	122.3 (7)	C36A—C31A—B2A	120.7 (7)
C31—C32—C33	121.0 (7)	C31A—C32A—C33A	122.1 (6)
C31—C32—S2	124.9 (6)	C31A—C32A—S2A	123.5 (5)
C33—C32—S2	114.1 (5)	C33A—C32A—S2A	114.4 (5)
C34—C33—C32	120.3 (8)	C34A—C33A—C32A	119.1 (7)
C34—C33—H33	119.9	C34A—C33A—H33A	120.4
C32—C33—H33	119.9	C32A—C33A—H33A	120.4
C35—C34—C33	120.9 (9)	C33A—C34A—C35A	120.8 (7)
C35—C34—H34	119.5	C33A—C34A—H34A	119.6
C33—C34—H34	119.5	C35A—C34A—H34A	119.6
C34—C35—C36	119.5 (8)	C36A—C35A—C34A	119.4 (7)
C34—C35—H35	120.3	C36A—C35A—H35A	120.3
C36—C35—H35	120.3	C34A—C35A—H35A	120.3
C35—C36—C31	121.2 (8)	C35A—C36A—C31A	122.5 (7)
C35—C36—H36	119.4	C35A—C36A—H36A	118.8
C31—C36—H36	119.4	C31A—C36A—H36A	118.8
			
O1—B1—C1—C2	−175.6 (7)	B1A—O1A—B2A—C21A	97.4 (18)
C11—B1—C1—C2	2.9 (11)	B1A—O1A—B2A—C31A	−83.6 (19)
O1—B1—C1—C6	6.4 (12)	O1A—B1A—C1A—C2A	−176.8 (6)
C11—B1—C1—C6	−175.2 (7)	C11A—B1A—C1A—C2A	2.5 (10)
C6—C1—C2—C3	−1.7 (10)	O1A—B1A—C1A—C6A	1.6 (11)
B1—C1—C2—C3	−179.8 (7)	C11A—B1A—C1A—C6A	−179.1 (7)
C6—C1—C2—S1	179.7 (6)	C6A—C1A—C2A—C3A	−1.5 (11)
B1—C1—C2—S1	1.6 (10)	B1A—C1A—C2A—C3A	176.9 (8)
C12—S1—C2—C1	−5.4 (7)	C6A—C1A—C2A—S1A	−179.2 (6)
C12—S1—C2—C3	176.0 (5)	B1A—C1A—C2A—S1A	−0.8 (10)
C1—C2—C3—C4	0.5 (11)	C12A—S1A—C2A—C1A	−2.3 (7)
S1—C2—C3—C4	179.3 (6)	C12A—S1A—C2A—C3A	179.9 (7)
C2—C3—C4—C5	0.5 (12)	C1A—C2A—C3A—C4A	3.0 (14)
C3—C4—C5—C6	−0.3 (12)	S1A—C2A—C3A—C4A	−179.0 (8)
C4—C5—C6—C1	−0.9 (13)	C2A—C3A—C4A—C5A	−3.0 (16)
C2—C1—C6—C5	1.9 (11)	C3A—C4A—C5A—C6A	1.6 (16)
B1—C1—C6—C5	−179.9 (8)	C4A—C5A—C6A—C1A	−0.2 (14)
O1—B1—C11—C12	176.1 (7)	C2A—C1A—C6A—C5A	0.1 (11)
C1—B1—C11—C12	−2.4 (11)	B1A—C1A—C6A—C5A	−178.3 (8)
O1—B1—C11—C16	−4.3 (12)	O1A—B1A—C11A—C12A	178.9 (6)
C1—B1—C11—C16	177.3 (8)	C1A—B1A—C11A—C12A	−0.5 (10)
C16—C11—C12—C13	−0.2 (10)	O1A—B1A—C11A—C16A	−2.0 (11)
B1—C11—C12—C13	179.4 (7)	C1A—B1A—C11A—C16A	178.7 (7)
C16—C11—C12—S1	177.8 (6)	C16A—C11A—C12A—C13A	−0.3 (11)
B1—C11—C12—S1	−2.5 (10)	B1A—C11A—C12A—C13A	178.9 (8)
C2—S1—C12—C11	5.8 (7)	C16A—C11A—C12A—S1A	177.6 (6)
C2—S1—C12—C13	−176.1 (6)	B1A—C11A—C12A—S1A	−3.2 (10)
C11—C12—C13—C14	0.3 (12)	C2A—S1A—C12A—C11A	4.3 (7)
S1—C12—C13—C14	−177.9 (7)	C2A—S1A—C12A—C13A	−177.6 (7)
C12—C13—C14—C15	−2.2 (13)	C11A—C12A—C13A—C14A	1.6 (16)
C13—C14—C15—C16	4.0 (14)	S1A—C12A—C13A—C14A	−176.5 (11)
C14—C15—C16—C11	−3.9 (14)	C12A—C13A—C14A—C15A	−1 (2)
C12—C11—C16—C15	2.0 (12)	C13A—C14A—C15A—C16A	−2 (2)
B1—C11—C16—C15	−177.6 (8)	C14A—C15A—C16A—C11A	2.9 (17)
O1—B2—C21—C22	−178.3 (7)	C12A—C11A—C16A—C15A	−1.8 (12)
C31—B2—C21—C22	0.9 (11)	B1A—C11A—C16A—C15A	178.9 (9)
O1—B2—C21—C26	1.5 (12)	O1A—B2A—C21A—C22A	177.1 (6)
C31—B2—C21—C26	−179.4 (7)	C31A—B2A—C21A—C22A	−2.0 (10)
C26—C21—C22—C23	−1.0 (10)	O1A—B2A—C21A—C26A	−2.4 (11)
B2—C21—C22—C23	178.8 (8)	C31A—B2A—C21A—C26A	178.5 (7)
C26—C21—C22—S2	−179.2 (6)	C26A—C21A—C22A—C23A	1.1 (10)
B2—C21—C22—S2	0.6 (10)	B2A—C21A—C22A—C23A	−178.4 (7)
C32—S2—C22—C21	−1.8 (7)	C26A—C21A—C22A—S2A	−177.9 (6)
C32—S2—C22—C23	179.9 (6)	B2A—C21A—C22A—S2A	2.6 (9)
C21—C22—C23—C24	1.7 (12)	C32A—S2A—C22A—C21A	−2.6 (7)
S2—C22—C23—C24	−179.9 (7)	C32A—S2A—C22A—C23A	178.4 (5)
C22—C23—C24—C25	−0.2 (14)	C21A—C22A—C23A—C24A	−0.3 (11)
C23—C24—C25—C26	−2.0 (15)	S2A—C22A—C23A—C24A	178.8 (6)
C24—C25—C26—C21	2.9 (15)	C22A—C23A—C24A—C25A	−0.9 (12)
C22—C21—C26—C25	−1.4 (13)	C23A—C24A—C25A—C26A	1.2 (12)
B2—C21—C26—C25	178.8 (9)	C24A—C25A—C26A—C21A	−0.4 (12)
O1—B2—C31—C32	178.5 (6)	C22A—C21A—C26A—C25A	−0.8 (11)
C21—B2—C31—C32	−0.7 (10)	B2A—C21A—C26A—C25A	178.7 (8)
O1—B2—C31—C36	−5.0 (11)	O1A—B2A—C31A—C32A	−177.2 (6)
C21—B2—C31—C36	175.8 (7)	C21A—B2A—C31A—C32A	1.8 (10)
C36—C31—C32—C33	1.6 (9)	O1A—B2A—C31A—C36A	1.8 (11)
B2—C31—C32—C33	178.3 (7)	C21A—B2A—C31A—C36A	−179.2 (7)
C36—C31—C32—S2	−177.5 (6)	C36A—C31A—C32A—C33A	1.0 (10)
B2—C31—C32—S2	−0.9 (9)	B2A—C31A—C32A—C33A	−180.0 (7)
C22—S2—C32—C31	1.9 (6)	C36A—C31A—C32A—S2A	178.7 (5)
C22—S2—C32—C33	−177.2 (6)	B2A—C31A—C32A—S2A	−2.2 (9)
C31—C32—C33—C34	0.5 (11)	C22A—S2A—C32A—C31A	2.3 (6)
S2—C32—C33—C34	179.7 (7)	C22A—S2A—C32A—C33A	−179.8 (5)
C32—C33—C34—C35	−1.2 (13)	C31A—C32A—C33A—C34A	−0.9 (10)
C33—C34—C35—C36	−0.2 (13)	S2A—C32A—C33A—C34A	−178.8 (6)
C34—C35—C36—C31	2.4 (13)	C32A—C33A—C34A—C35A	1.2 (11)
C32—C31—C36—C35	−3.1 (11)	C33A—C34A—C35A—C36A	−1.6 (12)
B2—C31—C36—C35	−179.7 (8)	C34A—C35A—C36A—C31A	1.8 (12)
B2A—O1A—B1A—C11A	171.1 (16)	C32A—C31A—C36A—C35A	−1.4 (11)
B2A—O1A—B1A—C1A	−10 (2)	B2A—C31A—C36A—C35A	179.5 (7)
