# Supplementary figures and images for: Polymorphism and pseudosymmetry of 10,10′-oxybis(9-thia-10-hydro-10-boraanthracene)
Source: Acta Crystallogr E Crystallogr Commun. 2019 Apr 25;75(Pt 5):690–4. doi: 10.1107/S205698901900522X (PMC6505595; doi:10.1107/S205698901900522X)

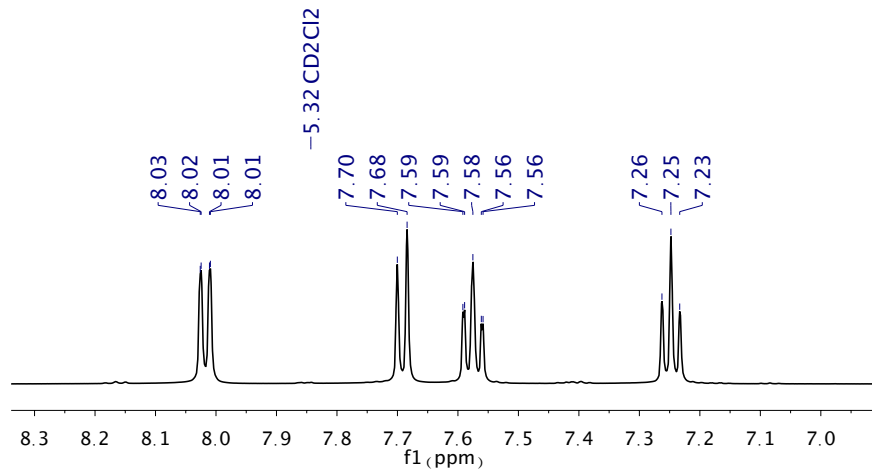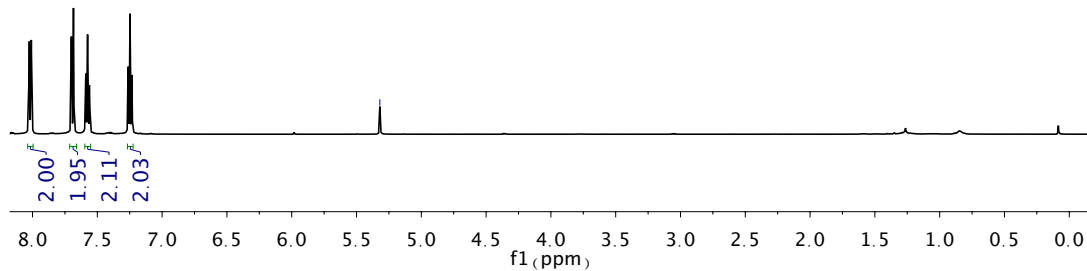

Supplement: Supplementary file 5 [file e-75-00690-sup5.pdf]

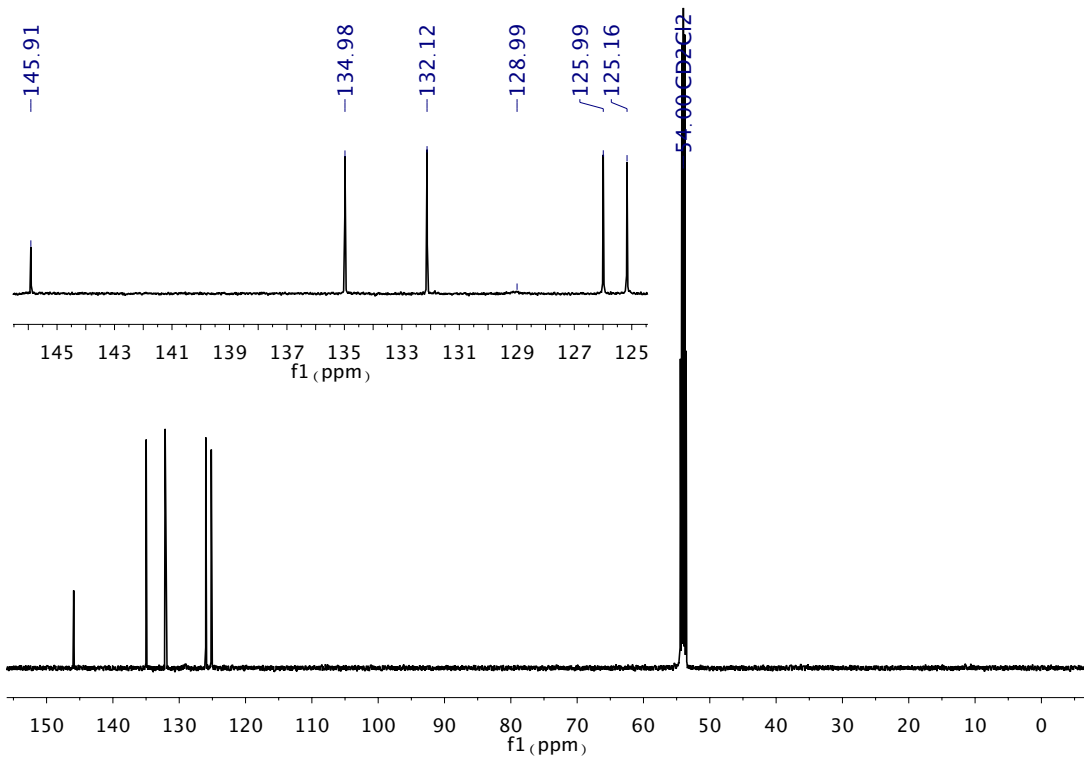

Supplement: Supplementary file 6 [file e-75-00690-sup6.pdf]

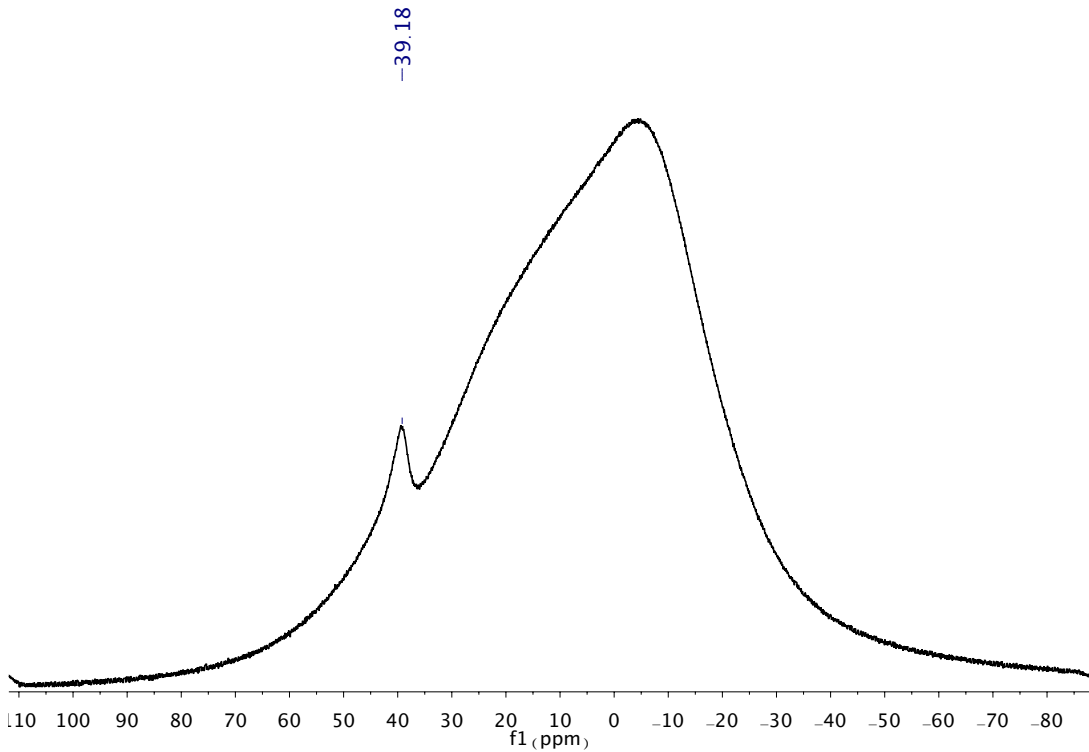

Supplement: Supplementary file 7 [file e-75-00690-sup7.pdf]
